# The multisensory cocktail party problem in adults: The effects of the configural talking-face template and of facial, vocal, and linguistic identity cues on its solution

**DOI:** 10.1371/journal.pone.0354673

**Published:** 2026-07-28

**Authors:** David J. Lewkowicz, Julia McClellan

**Affiliations:** 1 Child Study Center, Yale University School of Medicine, New Haven, Connecticut, United States of America; 2 Department of Psychology, Yale University, New Haven, Connecticut, United States of America; University of Missouri Columbia, UNITED STATES OF AMERICA

## Abstract

Social communication often involves competing talkers and, thus, gives rise to the multisensory cocktail party problem (MCPP). To solve the MCPP, perceivers must bind, integrate, and segregate each talker’s auditory (A) and visual (V) speech streams. Audiovisual (AV) temporal synchrony is a known and powerful multisensory segregation cue and facial and vocal identity cues are known to facilitate segregation. Using eye tracking, we investigated whether perceptual segregation of multiple talking faces depends on the configural talking-face template (Experiment 1) and whether facial, vocal, and linguistic identity cues associated with different talkers can facilitate perceptual segregation (Experiment 2). Results indicated (a) that AV synchrony-based perceptual segregation of multiple talking faces does not depend on the configural talking-face template, (b) that linguistic identity cues facilitated synchrony-based segregation of upright talking faces independently of facial and vocal identity cues, (c) that perceptual segregation was driven primarily by AV speech processing as evidenced by greater selective attention to the talkers’ mouth, and (d) that, as evidenced by pupillary dilation, the type and number of AV congruence cues that had to be processed for successful segregation was associated with differential cognitive effort. Overall, these findings add to existing evidence indicating that adults’ solution of the MCPP relies on AV temporal synchrony cues and on facial, vocal, and linguistic identity congruence cues but not on the canonical configural talking-face template.

## Introduction

Most social events involve multiple people talking at the same time. Such events are cluttered multisensory scenes specified by individuals represented by unique facial and vocal attributes producing linguistically distinct streams of fluent A and V speech. To communicate effectively with one another, the participants at such events must solve what Lewkowicz, Schmuckler, and Agrawal [1,2] have referred to as the Multisensory Cocktail Party Problem (MCPP). To solve the MCPP, perceivers must rapidly search the multiple talker scene, detect the multiple A and V speech streams, bind them into their corresponding pairs, integrate them into unified multisensory entities, and segregate them into unitary and meaningful multisensory representations of different talkers.

### Mechanisms of perceptual segregation of multiple talking faces

In a multi-talker scene, the binding and integration of each talker’s A and V speech streams is a necessary pre-requisite for the perceptual segregation of the different talkers. If so, what perceptual cues might facilitate such binding and integration and the ensuing perceptual segregation of the unitary multisensory representations of each talker? Several cues are likely candidates. These include: (a) the temporally synchronized dynamics of each talker’s A and V speech streams, (b), the canonical orientation of talkers’ faces and (c) the unique facial, vocal, and linguistic identity cues that represent each talker.

### AV temporal synchrony

AV temporal synchrony is a low-level, domain-general feature of the multisensory world and its perception does not depend on learning nor on sophisticated neural computations [3–6]. Therefore, it is not surprising that perceptual sensitivity to AV temporal synchrony is present in infancy and that it plays a major role in the developmental emergence of unified multisensory representations of our internal and external worlds and that it enables us to have veridical perceptual experiences [4,7–12]. Of course, as development progresses, higher-level multisensory congruence cues are gradually discovered and this enables us to create more complex multisensory representations [3]. Crucially, however, AV temporal synchrony continues to play a major role in multisensory binding, integration, and the creation of multisensory representations right into adulthood [3–6,13–17].

### Canonical configural talking-face template

The canonical orientation of a talking face may play a role in the perceptual segregation of multiple talking faces because a change in face orientation leads to a change in the spatial position and relationships of the visible articulators as well as in a violation of the mental prototype for visual speech [18]. The mental prototype for V speech is the product of extensive developmental experience with upright faces, in general, and with upright talking faces, in particular. This experience begins at birth and quickly – over the first few months – leads to the emergence of a template for the canonical orientation of a silent face [19,20] as well as a talking face [21–28]. Especially important in the context of the MCPP, the template for the canonical talking face specifies the spatial location of the deictic and social cues needed for social interaction (i.e., the eyes) and the spatial location of AV speech used in linguistic communication (i.e., the mouth).

The canonical configural talking-face template emerges at around six months of age, and once it does, infants discover the source of AV speech. This is especially advantageous for the development of speech, language, and communication because AV speech, as opposed to A speech, is perceptually more salient. This is evident in findings from adult studies showing that comprehension of redundantly specified AV speech is generally more robust than comprehension of A speech or A speech presented in noise [29–31]. Indeed, once infants discover the greater salience of redundantly specified AV speech, they begin relying on it by resorting to lipreading whenever they need to disambiguate unfamiliar, non-native speech [21,22]. Then, as development progresses, lipreading becomes the default mode whenever speech processing becomes challenging. This is illustrated by findings that children resort to lipreading when they need to disambiguate unfamiliar speech [32,33] or when they are exposed to noisy speech [34] and by findings that adults resort to lipreading when they need to process either non-native speech or speech presented in noise [35–38].

Interestingly, even though the canonical face and the canonical talking-face templates are well established in adulthood, evidence of their importance for perception has been mixed. On the one hand, it is known that face inversion disrupts holistic face processing while it preserves part-based analytical face processing in both children [39] and adults [40–42]. On the other hand, it is also known that face inversion does not disrupt the processing of AV syllables [18,43–45]. What is not known, however, is whether face inversion disrupts the processing of fluent AV speech, especially in the context of the MCPP. One possibility is that the temporally synchronized dynamics of fluent AV speech play an outsize role in the processing of talking faces. Therefore, if face inversion mostly disrupts the holistic/configural processing of faces, it may be that the perceptual power of AV temporal synchrony cues to facilitate multisensory binding, integration, and segregation may be preserved even when talking faces are in their non-canonical spatial orientation.

### AV identity cues

Behavioral [46–48] and neural [49] evidence indicates that adults can associate the A and V identity attributes of individual talkers. Consistent with this fact, Lewkowicz, et al. [1] found that the unique facial and vocal identity cues that usually represent different talkers also can facilitate multisensory segregation. Currently, however, it is not known whether the AV linguistic identity cues that are usually associated with different talkers also might contribute to multisensory segregation of multiple talkers. It is highly likely that they do given that the unique linguistic productions of different talkers typically consist of distinct sets of prosodic, phonetic, lexical, syntactic, and semantic perceptual attributes that, together, can give rise to distinct multisensory representations.

### Multisensory redundancy and its significance for the MCPP

As already indicated, redundantly specified AV speech is generally more salient and, as a result, its perception is more robust than the perception of A speech. Importantly, the advantage that redundantly specified AV speech provides reflects a more general property of redundantly specified multisensory inputs in general. That is, findings from many studies using simple to complex stimuli have demonstrated that redundantly specified multisensory inputs yield more robust detection, learning, and memory than do unisensory inputs [3,31,50–62].

The advantage of multisensory redundancy is especially important for the MCPP because the greater perceptual salience of redundantly specified AV inputs facilitates search behavior, a process critical to solving the MCPP. This is illustrated by studies of search behavior of cluttered scenes composed of simple objects and a sound. These studies have found that the search for a target object is faster and more efficient when its actions are accompanied by a temporally synchronized sound [57,61,63,64]. Similarly, it has been found that the perceptual segregation of AV speech is faster and more robust than the search of A-only speech [58,65] and that the tracking of the speech envelope of an utterance is facilitated when a speaker’s face can be seen at the same time [66].

### Perceptual segregation of multiple talking faces: the MCPP paradigm

In a pair of studies, one with adults and one with children, Lewkowicz et al. [1,2] introduced a new experimental paradigm specifically designed to investigate the perceptual segregation of multiple talking faces as a proxy for studying the MCPP. The primary question in these initial studies was whether AV temporal synchrony and facial and vocal identity contribute to the perceptual segregation of multiple talking faces.

In Experiment 1 of the Lewkowicz et al. [1] study, the question was whether adults can take advantage of AV temporal synchrony cues to bind and integrate the A and V attributes of multiple talking faces and perceptually segregate them once they are integrated. To answer this question, participants saw four identical talking faces in the four quadrants of a screen speaking the same utterance. While they watched the talking faces, they also heard the A version of the V utterance. In the synchrony condition, the A utterance was temporally synchronized with one of the four talking faces and desynchronized from the other 3 talking faces, whereas in the asynchrony condition, the A utterance was desynchronized from all four talking faces. Measures of eye gaze, which served as an index of selective attention, indicated that participants exhibited a marked preference for the target talking face in the synchrony condition and no such preference in the asynchrony condition. These findings demonstrated that AV temporal synchrony plays a major role in AV binding and integration and, ultimately, in the perceptual segregation of integrated multisensory entities.

In Experiment 2 of the Lewkowicz et al. [1] study, the question was whether adults can take advantage of facial and vocal identity cues, in addition to AV temporal synchrony cues, in AV binding, integration, and perceptual segregation of multiple talking faces. To answer this question, participants saw four different talking faces in the four quadrants of a screen all speaking the same utterance and heard the A version of the V utterance produced by one of them. Because the four talking faces were now different, the vocal and prosodic signatures of each talker’s utterances were unique. Like in Experiment 1, the A utterance was temporally synchronized with one of the four talking faces and desynchronized from the other 3 talking faces in the synchrony condition but desynchronized from all four talking faces in the asynchrony condition. Given that each talking face was now different, that its V prosodic signature was different, and that each talker’s vocal and A prosodic identity cues were distinct, preferences could now be based on AV temporal synchrony as well as the unique V and A identity cues associated with each talker. Indeed, now participants exhibited two preferences. One was a marked preference for the temporally synchronized talking face in the synchrony condition, replicating the findings from Experiment 1 in the Lewkowicz et al. [1] study. The other was a preference for one of the temporally desynchronized talking faces in the asynchrony condition which, in this experiment, was still congruent with the A utterance in terms of identity cues.

Examination of selective attention to the eyes and mouth revealed that attention to the mouth was substantially greater than to the eyes in both experiments. This indicated that participants complied with the task demand to find the talking face by focusing most of their attention on the source of AV speech and processing it to determine whether the A and V information was congruent (i.e., whether it matched). When it was congruent, they manifested this with a preference for that talking face. Crucially, this speech-processing interpretation is consistent with findings from previous studies of AV speech processing [21,35,38,67] and with the fact that participants attended more to the mouth when the V speech utterance of none of the talking faces was temporally synchronized with the A speech utterance. Presumably, the absence of a talking face whose V and A speech utterances were congruent required more attention to the source of AV speech to ensure that the talking face was not missed.

### Current study and predictions

The Lewkowicz et al. [1] findings demonstrate that AV temporal synchrony plays a powerful role in multisensory binding, integration, and segregation of a multi-talker scene and that facial and vocal identity cues facilitate these processes. These findings raise three questions. First, given that the talking-face template emerges early in life, does the detection of the temporal synchrony of the dynamics inherent in fluent A and V speech streams depend on the canonical orientation of talkers’ faces? Second, given that unique linguistic cues are usually associated with different talkers’ AV speech productions, might these cues also facilitate multisensory binding, integration, and segregation? Finally, does the presence vs. absence of an audiovisually congruent talking face require different amounts of cognitive effort to comply with task demands to find the talking face and does the type and number of multisensory congruence cues affect the cognitive effort involved in finding the audiovisually congruent talking face?

To answer these questions, we conducted two experiments by using the MCPP experimental paradigm first introduced by Lewkowicz et al. [1]. To reiterate, the essential elements of the MCPP paradigm are the presentation of 4 talking faces in the 4 quadrants of a screen. Participants can simultaneously see the 4 faces talking and hear an A utterance. During the synchrony condition, the A utterance is temporally synchronized with one of the talking faces while during the asynchrony condition it is desynchronized from all 4 talking faces. Using eye tracking, participants’ gaze to areas-of-interest (AOIs), including each face and the eyes and mouth of each face, is measured as a proxy for determining how selective attention to the different AOIs is affected by different perceptual cues when the participants’ assigned task is to identify “the talking face”.

**Fig 1:**
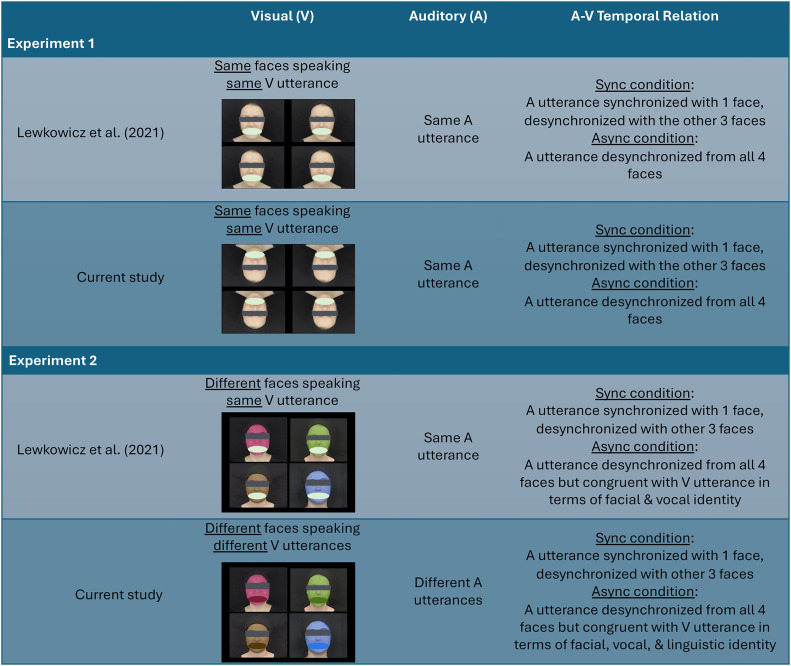
Stimuli presented in Experiments 1 and 2 in the Lewkowicz et al. (2021) and in the current study. The video stimuli presented in the Lewkowicz et al. (2021) study can be viewed at https://doi.org/10.1016/j.cognition.2021.104743 and the video stimuli presented in the current study can be viewed at https://osf.io/swzat/?view_only=e571661179694ec4a8cf1a8817af6b64. The V utterances all began at the same time but were temporally jittered with respect to one another, meaning that the starting point of 3 of the 4 V utterances was increasingly later into the utterance relative to the starting point of the first V utterance.

We used the MCPP paradigm in Experiment 1 by replicating Experiment 1 in the Lewkowicz et al. [1] study. The one critical difference was that here we presented the four identical talking faces in an inverted position to determine whether the canonical talking-face template plays a role in the solution of the MCPP. We also used the MCPP paradigm in Experiment 2 to investigate the possible differential contribution of facial, vocal, and AV linguistic identity cues to the binding, integration, and segregation of multiple talking faces. In this case, Experiment 2 was a direct extension of Experiment 2 in the Lewkowicz et al. [1] study in that, here, participants saw also saw four different talkers except that now they saw them speaking a different utterance and heard one of the 4 distinct A utterances across test trials presented either in synchrony with the matching talking face or desynchronized from all 4 talking faces. The primary dependent measure was selective attention (i.e., gaze) directed to the face, eye, and mouth AOIs while a secondary dependent measure was pupil diameter as a proxy for cognitive effort. To facilitate cross-study comparisons with the similar experiments reported by Lewkowicz et al. [1], Fig 1 provides details about the specific stimuli presented in the current study and in the Lewkowicz et al. [1] study.

In Experiment 1, we expected to find a marked preference for the audiovisually congruent talking face in the synchrony condition and no preference in the asynchrony condition. This prediction is based on the earlier-cited evidence that AV temporal synchrony cues play a powerful role in perception in that they enable the binding and integration of multisensory inputs and, in the process, render multisensory inputs perceptually more salient than unisensory inputs. With specific regard to the AV speech domain, fluent AV speech consists of temporally dynamic streams of A and V speech streams that, when bound and integrated, are perceptually more salient than unisensory A streams and, most likely, more salient than unisensory and relatively static facial configuration and orientation cues. As a result, it is likely that AV temporal synchrony cues are perceptually primary when they compete with facial configuration and orientation cues and, thus, that participants will exhibit a preference for the audiovisually congruent talking face even when the talking faces are inverted. In Experiment 2, we expected to find a marked preference for the audiovisually congruent talking face in the synchrony condition as well as a preference for the audiovisually desynchronized virtual target talking face. The latter preference is expected given that the virtual target face is congruent with the A utterance in terms of its facial, vocal, and linguistic identity cues. In addition to the face preferences, we also expected that, in both experiments, participants would direct more attention to the mouth than the eyes and that they would do so more when an audiovisually congruent talking face was not present in the stimulus array than when it was present. Finally, we expected to find greater pupil dilation (i.e., greater cognitive effort) in the asynchrony condition relative to the synchrony condition in both experiments and greater pupil dilation when participants must process more multisensory congruence cues (i.e., in Experiment 2) than when they have to process less of them (i.e., in Experiment 1).

## Experiment 1

To investigate whether the perceptual extraction of the dynamic information specified by fluent AV speech depends on the canonical orientation of faces specified by our developmentally acquired talking-face template, we presented four identical talking faces in an inverted orientation. Each face spoke the same utterance. Crucially, however, the visible utterances were presented in a temporally jittered manner with respect to one another, meaning that the starting point of the visible utterances occurred increasingly later into the utterance across the four visible utterances. One result of the temporal jittering was that it seemed as if each talking face was producing a different V utterance. A second result of the temporal jittering was that it made it possible to temporally synchronize the A version of the same utterance with one of the four talking faces and desynchronize it from the other three talking faces. As indicated above, we expected to obtain evidence that adults can extract the dynamics of fluent V speech as easily from inverted faces as from upright faces and that they can use this information to solve the MCPP.

## Methods

### Participants

We tested 27 adults (20 females) who ranged between 18.7 and 48.2 years of age (mean age = 22.5 years, SD = 6.3 years). The recruitment for this experiment started on February 5, 2022 and ended on April 13, 2022. All procedures involving human participants were approved by the Yale University Institutional Review Board (Protocol #2000026034). All participants were English-speaking volunteers who gave their written informed consent prior to taking part in the study.

### Apparatus and Stimuli

The stimuli were presented on a Dell Precision M4800 laptop computer screen (11 x 13-inch) and attention to the stimuli was measured with a REDn SensoMotoric Instruments (SMI, Teltow, Germany) remote eye tracker running at a sampling rate of 60 Hz and attached to the bottom of the computer’s screen. SMI’s iViewRed software controlled the eye tracker camera and processed the eye gaze data while SMI’s Experiment Center software-controlled stimulus presentation and data acquisition. Participants sat in front of the laptop with their eyes approximately 60 cm from the eye tracker’s camera. The A stimuli were presented through Sony Professional headphones (Model # MDR-7506) at a comfortable listening level. Testing took place in a quiet room.

The experiment began with a calibration phase and then continued with the presentation of two 15 s practice trials followed by thirty-two 15 s test trials. To calibrate eye gaze, a small yellow star was presented in the center of the screen as well as in each of the four corners of the screen. The stimuli used for the practice and for the experimental trials were the same composite videos that were presented by Lewkowicz et al. [1]. They consisted of four identical talking faces shown in each of the four equally sized quadrants (see Fig 1). In contrast to Lewkowicz et al. [1], however, here the four talking faces were presented in an inverted orientation. The female actor presented in the composite videos shown during the practice trials was different from the two female actors who appeared in the composite videos presented during the test trials. The V speech utterances articulated by all four talkers and the concurrently presented A utterance were identical.

The experiment consisted of 16 synchrony and 16 asynchrony test trials. In the synchrony test trials, the A speech utterance was temporally synchronized with the V speech utterance produced by one of the four talkers (the target) and desynchronized from the V speech utterances produced by the other three talkers (the distractors). In the asynchrony trials, the A speech utterance was desynchronized from all four V speech utterances.

We constructed four different sets of test trials by filming each of two test-phase female actors speaking two different sets of two different utterances. The four utterances were as follows: [1] “But your favorite will be the elephants. They’re big and gray and have large floppy ears. Maybe we’ll see a baby elephant too? What do you think about that? If not, we could go to story time at the library. All your friends will be there”; [2] “They like to ice skate, right? But, before we can go anywhere, what do we have to do? Change your clothes and eat breakfast, of course. It’s cold outside, so you need to wear a sweater. How about the green one with the duck? For breakfast, you can have oatmeal with blueberries.”; [3] “Good morning, get up, come on now. If you get up right away, we’ll have an hour to play in the house. I love these long mornings, don’t you. I wish they could last all day.”; [4] “We can hang around all day Saturday. Except, of course, for the party. Are you going to help me fix up the house? Are you? We need to buy flowers, prepare the food, vacuum the house, dust.”

Each set of test trials included four synchrony and four asynchrony trials. The synchrony test trials were composed of one target stimulus and three distractor stimuli and the target stimulus was presented in each of the four quadrants across trials. The asynchrony trials were identical to the synchrony trials except that the target face was audiovisually desynchronized. To determine whether AV temporal synchrony affected responsiveness, we compared gaze to the talking face presented in each quadrant, respectively, across the two synchrony conditions. Because the talking face in the asynchrony condition was audiovisually desynchronized, we designated it the “virtual” target to distinguish it from the corresponding audiovisually synchronized talking face presented in the same quadrant in the synchrony condition.

The composite videos were created and edited in Premiere Pro (Adobe Systems, Inc., San Jose, CA). They were constructed by first combining four identical audiovisually synchronized videos of each actor into a single video composed of an audiovisually synchronized talking face (the target) and three audiovisually desynchronized talking faces (the distractors). Crucially, the distractors’ V speech streams also were desynchronized with respect to each other through temporal jittering. This meant that the start point of the visible articulations produced by each distractor face was delayed increasingly later into the utterance relative to the start point of the V articulation produced by the target face. Consequently, the V speech stream articulated by each distractor face was temporally delayed with respect to the A speech stream by a fixed interval of time. The intervals for the two utterances spoken by one of the actors were 2200, 3300, and 4400 ms in both the synchrony and asynchrony test trials while the interval for the asynchronous version of the target stimulus in the asynchrony trials was 1800 ms. The intervals for one of the utterances spoken by the second actor were 1966, 2966, and 3899 ms in the synchrony and asynchrony test trials while the interval for the asynchronous version of the target stimulus in the asynchrony trials was 1799 ms. The intervals for the second utterance spoken by the second actor were 966, 1766, and 2633 ms in the synchrony and asynchrony test trials while the interval for the asynchronous version of the target in the asynchrony trials was 2933 ms. Importantly, even though some of intervals separating the V and A streams differed by less than 1 s, the individual videos were perceptually different from one another. Also, despite starting at different relative points of the utterance, the V speech articulations of all four talking faces began simultaneously at the start of each test trial. Sample stimulus videos presented in this study can be found at https://osf.io/swzat/?view_only=e571661179694ec4a8cf1a8817af6b64.

Procedure. The experimenter sat next to the participants, monitored the experiment, and answered any questions. All eye tracking data were acquired from the right eye. The experiment began with the calibration routine and calibration was deemed acceptable if the point of fixation fell within less than 1° of visual angle of the star’s position. Following calibration, written instructions were presented on the screen and participants were invited to ask questions.

The experiment commenced with two practice trials during which participants were familiarized with the procedure and were given the following instructions: “You will see four faces on the screen and hear a voice talking. Please look carefully to determine which face is talking.” The start of each new trial was under the participants’ control and was implemented by asking them to look at a continuously looming/receding yellow disc which appeared in the center of the screen after the end of each trial. As soon as they looked at the disk, the new trial started. The composite videos presented during the practice trials were composed of four identical videos of the same person articulating the same V utterance together with the A version of the same utterance. The A utterance was synchronized with the V speech utterance produced by one of the talking faces in the first practice trial but desynchronized from all four talking faces during the second practice trial. After each practice trial, a composite video of the same four faces was presented again except that this time the faces were still. Participants were asked to look at these still faces and indicate which one corresponded to the talking face in the immediately preceding composite video by pressing the number 1, 3, 7, or 9 on the laptop’s numerical keypad. These numbers were spatially congruent with the four quadrants in which the talking faces in the immediately preceding video appeared. Once participants completed the practice trials, they were given a chance to ask questions again and then the experiment proper began.

To control for order effects, the 32 test trials that followed were presented according to one of four randomly generated test trial orders. Participants were randomly assigned to one of four test groups that corresponded to the different orders. Trial randomization made it difficult to predict which specific actor or utterance would be presented on a given trial, in which quadrant the target face would appear, and whether the A speech utterance would be synchronized or not with the V speech produced by the target face.

Like in the practice trials, immediately following each test trial, we presented a composite still image of the same four faces that were presented during that test trial. Participants were asked to indicate which of these still faces corresponded to “the talking face” in the immediately preceding test trial. To do so, they were asked to press the numbers 1, 3, 7, or 9, corresponding to the spatial position of each face by using the numerical keypad. The primary purpose of the key presses was to induce the participants to pay attention and to comply with the assigned task of finding “the talking face”. As a result, we did not record their choices.

To measure selective attention, we created an area-of-interest (AOI) for each of the four faces and the eyes and mouth of each of those faces (see Fig 1). We used the total amount of looking directed at each AOI to derive two sets of dependent measures for each test trial. The first set consisted of the proportion of total looking time (PTLT) directed at each talking face. This PTLT was computed by dividing the total amount of looking at each respective face AOI by the total amount of looking at all four face AOIs. The second set of dependent measures consisted of the PTLT directed to the eyes and mouth of each respective face. This measure was computed by dividing the total amount of looking at the eyes and mouth of each respective face by the total amount of looking at that face.

In the Introduction, we hypothesized that participants may expend greater cognitive effort when searching for a missing audiovisually synchronized talking face in the asynchrony condition than when searching for one that is present in the synchrony condition. To determine if this was the case, we relied on the fact that pupil diameter generally increases as cognitive effort increases [68] and, therefore, compared pupil diameter across the two synchrony conditions. At a sampling rate of 60 Hz, the SMI eye tracker reported pupil diameter every 16.66 ms except for when an eye blink occurred or when the eye tracker was unable to measure pupil diameter and reported a null value. Using the available data collected during each 16.66 ms epoch of a given trial, we computed the average pupil diameter for each of the four face AOIs, separately for each trial. Then, we computed an average score across the four face AOIs per trial. In those few trials where participants either fixated only the target-face AOI or only the distractor-face AOIs, pupil diameter data were missing for the non-fixated face AOIs. In those cases, the missing data for the non-fixated AOI were replaced by the grand mean for the non-fixated AOI across all test trials for all participants. Out of 512 cells for the target-AOI, 10 were replaced for the synchrony condition and 16 for the asynchrony condition. Out of 512 cells for the average of the three distractor AOIs, 36 were replaced for the synchrony condition and 8 for the asynchrony condition.

*Statistical Analyses*. Our experimental design called for repeated measures on each participant. This resulted in a nested data structure that might have introduced individual difference effects. To rule out the possibility that individual differences may have unduly influenced responding, we analyzed the data with a Linear Mixed-Effects Model (LMM) implemented in the gamlj package (v. 2.7.24.0) in jamovi. The variance components were estimated using the Restricted Maximum Likelihood (REML) and the degrees-of-freedom were calculated with the Satterthwaite method. To conform with currently recommended best practices [69], we report the results of each LMM analysis in the Supporting Information section and, for each analysis, we include model information, model fit, fixed effects omnibus tests, parameter estimates for the fixed coefficients, and random components effects.

## Results

The principal question in this experiment was whether the canonical configural talking-face template plays a role in the extraction of AV temporal synchrony and whether it is necessary for perceptual segregation in the context of the MCPP. If it is not, then face inversion should not disrupt perceptual segregation and, thus, like in Experiment 1 in the Lewkowicz et al. [1] study, participants should exhibit a preference for the target talking face over the distractors in the synchrony condition and no preference in the asynchrony condition. Furthermore, participants should exhibit greater overall attention to the mouth than eyes and greater attention to the mouth when a synchronized talking face is not present in the stimulus array than when it is present.

### Face AOIs

*LMM Analysis of PTLT Scores*. Using PTLT scores as the dependent variable, we compared these scores for the target talking face with the average of the PTLT scores for the three distractor talking faces. To model the effects of the variables of interest as well as their interactions in LMM, we defined Synchrony Condition (2, Synchrony, Asynchrony) and Stimulus Type (2, Target, Distractor) as fixed effects and Subjects as random components.

**Fig 2:**
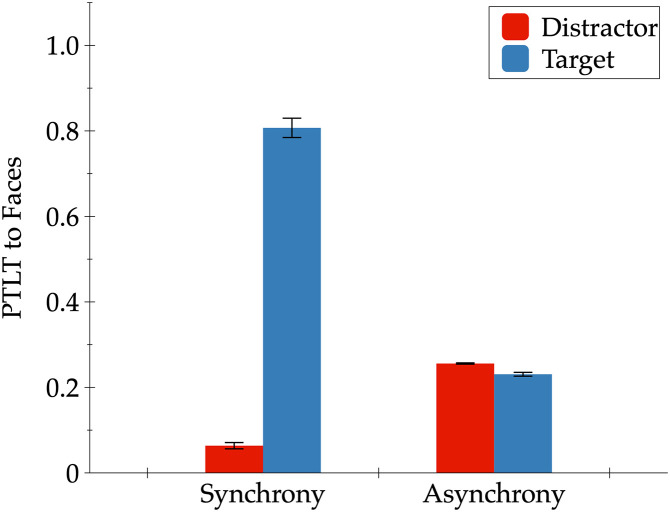
Selective attention to the temporally desynchronized (distractor) and synchronized (target) talking faces in the synchrony and asynchrony conditions in Experiment 1. Mean proportion of total looking time (PTLT) at the distractor and target faces across the two synchrony conditions. Error bars represent standard errors of the mean. The data underlying this figure can be found at https://osf.io/swzat/overview?view_only=e571661179694ec4a8cf1a8817af6b64.

The main effects of Synchrony Condition, *F*(1, 78) = 218.83, *p* < .001, and Stimulus Type, *F*(1, 78) = 764.03, *p* < .001, were statistically significant as was the interaction between these two variables, *F*(1, 78) = 876.80, *p* < .001. Fig 2 depicts the Synchrony Condition x Stimulus Type interaction and, as can be seen, participants looked much longer at the target face than at the distractor faces in the synchrony condition but not in the asynchrony condition. Simple effects analyses indicated that the 2-way interaction seen in Fig 2 was due to greater looking at the target talking face relative to looking at the distractor talking faces in the synchrony condition, *F*(1, 78) = 1623.46 *p* < .001, and the absence of such a difference in the asynchrony condition, *F*(1, 78) = 1.96, *p* = .166. Furthermore, the simple effects analyses showed that looking at the target talking face was greater than at the virtual target talking face, *F*(1, 78) = 995.00, *p <* .001, and that looking at the distractor faces was greater in the asynchrony than the synchrony condition, *F*(1, 78) = 108.75, *p <* .001. Thus, as predicted, even though the talking faces were inverted, participants successfully detected the AV temporal synchrony information inherent in the target talking face and they exhibited a preference for this face.

**Fig 3:**
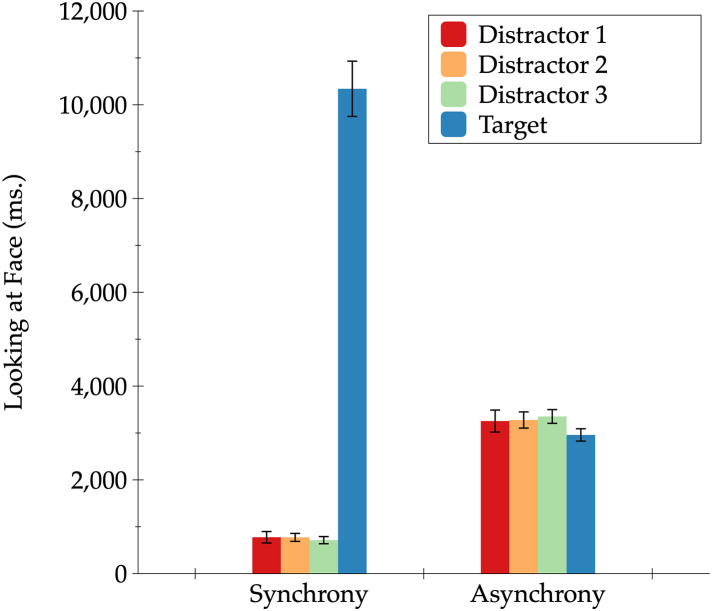
Selective attention to each distractor face as well as the target face across the two synchrony conditions in Experiment 1. Mean looking time in seconds directed to each distractor face as well as the target face in the synchrony and the asynchrony condition in Experiment 1. Error bars represent standard errors of the mean. The data underlying this figure can be found at https://osf.io/swzat/overview?view_only=e571661179694ec4a8cf1a8817af6b64.

*LMM Analysis of Raw Looking Time Scores.* The primary dependent variable in the current study was the PTLT score. Although such a score controls for possible individual differences in absolute looking times, it does not provide any insights into the relationship between PTLT scores and raw looking time scores. Ideally, PTLT scores and raw looking time scores should yield similar patterns of findings. As Fig 3 shows, this was the case in that participants attended far more to the target face than the distractor faces in the synchrony condition and that this was not the case in the asynchrony condition. An LMM analysis, with looking time in seconds as the dependent variable, Synchrony Condition (2, Synchrony, Asynchrony) and Stimulus (2, Distractor, Target) as fixed effects, and Subjects as random components, yielded a main effect of Stimulus, *F*(3, 3422) = 1281.60, *p <* .001, as well as a significant interaction of Stimulus x Synchrony Condition, *F*(3, 3422) = 1483.52, *p <* .001. To clarify the 2-way interaction, we conducted simple effects analyses. These yielded a significant stimulus effect in both the synchrony, *F*(3,3302) = 2748.89, *p* < .001, and the asynchrony, *F*(3,3302) = 3.85, *p* = .009, condition. In addition, they yielded greater looking at distractors 1, 2, and 3 in the asynchrony than the synchrony condition (*F*(1, 3422) = 371.28, *p* < .001; *F*(1, 3422) = 381.50, *p* < .001; and *F*(1, 3422) = 422.60, *p* < .001, respectively) as well as greater looking at the target in the synchrony than in the asynchrony condition, *F*(1, 3422) = 3276.32, *p* < .001. Overall, these findings indicate that the response pattern derived from PTLT scores essentially mirrored the pattern derived from raw looking time scores.

### Eye and Mouth AOIs

**Fig 4:**
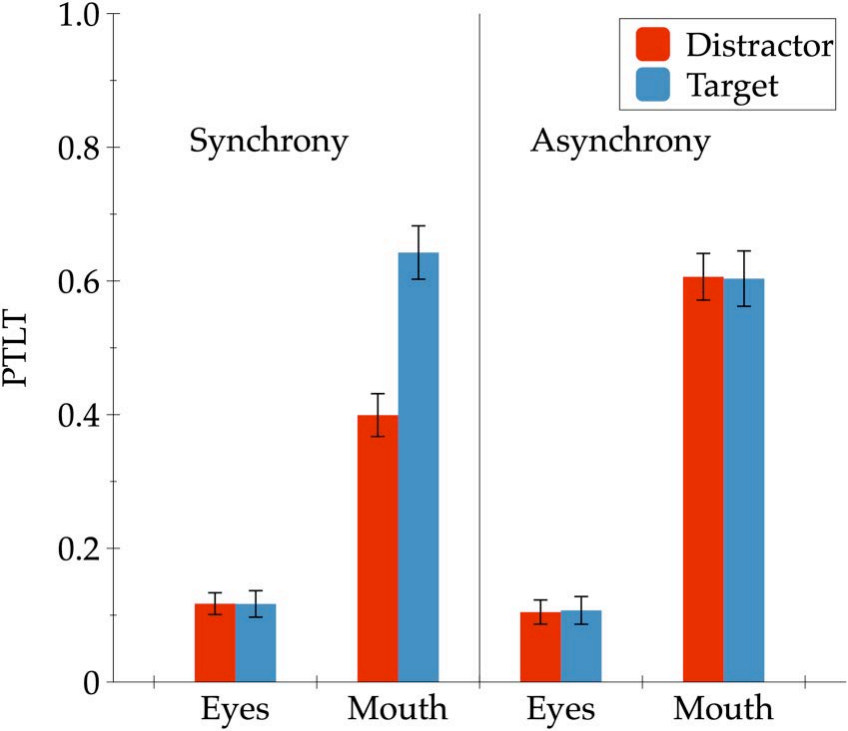
Selective attention to the eyes and mouth in Experiment 1. Mean proportion of total looking time to the eyes and mouth of the distractors and the target, respectively, in the two synchrony conditions in Experiment 1. Error bars represent standard errors of the mean. The data underlying this figure can be found at https://osf.io/swzat/overview?view_only=e571661179694ec4a8cf1a8817af6b64.

Using LMM, we modeled the effects of the fixed factors, namely Synchrony Condition (2, Synchrony, Asynchrony), Stimulus Type (2, Distractor, Target), and AOI (2, Eyes, Mouth) and Subjects as random components on PTLT scores. As Fig 4 shows, the main effect of AOI was significant due to a marked preference for the talker’s mouth, *F*(1, 182) = 501.26, *p* < .001, indicating that participants engaged in AV speech processing. The main effect of AOI was qualified by a significant Synchrony Condition x AOI x Stimulus Type interaction, *F*(1, 182) = 8.14, *p* = .005. This was due to different patterns of relative looking at the eyes vs. mouth as a function of stimulus type and the presence or absence of an audiovisually synchronized talking face. Simple effects analyses showed that looking at the mouth was greater in the asynchrony than synchrony condition, *F*(1, 182) = 8.45, *p* = .004, and that this, in turn, was due to greater looking at the mouth of the target than the distractor talking faces in the synchrony condition, *F*(1, 182) = 14.55, *p* < .001, but not in the asynchrony condition, *F*(1, 182) = 0.001, *p* = .978.

In sum, even though holistic face processing was disrupted by face inversion in this experiment, the mouth still captured far more attention than did the eyes. Interestingly, the target’s mouth captured more attention than did the distractors’ mouth when an audiovisually synchronized talking face was present in the stimulus array. In contrast, when an audiovisually synchronized talking face was not present in the stimulus array, the mouth of the virtual target and the distractors captured equal amounts of attention. This pattern of differential attention to the mouth across the synchrony conditions suggests that the audiovisually synchronized talking face popped out of the stimulus array and that, once it did, there was less need to examine the distractor faces to determine if one of them was audiovisually synchronized or not.

### Cognitive Effort

Earlier, we hypothesized that a search for an audiovisually synchronized talking face is likely to elicit less cognitive effort when it is present in the stimulus array than when it is not and that this should be evident in pupil size differences [68]. We based this hypothesis on the assumption that the perceptual pop-out effect found here as well as in previous studies of multisensory search [1,57,61] facilitates the search. Therefore, using LMM, we modeled the effects of trial and synchrony condition on pupil diameter, with Trial [16] and Synchrony Condition (2, Synchrony, Asynchrony) as fixed effects and Subjects as random components. Results yielded a significant Trial effect, *F*(15, 961) = 5.10, *p* < .001, a non-significant Synchrony Condition effect, *F*(1, 961) = 1.82, *p* = .177, and a significant Trial x Synchrony Condition interaction, *F*(15, 961) = 2.48, *p* = .001. The Trial effect reflects a decrease in pupil diameter over the course of the experiment and it is not surprising given that this is a common occurrence in studies measuring pupil diameter [70]. In the present case, the decline most likely reflects a combination of fatigue and decreasing cognitive engagement. Contrary to expectation, cognitive effort did not differ across the two synchrony conditions. Nonetheless, it should be noted that the significant Trial x Synchrony Condition interaction suggests that there was perhaps a subtle but more complex effect of synchrony condition on cognitive effort.

## Discussion

We found that adults are capable of multisensory binding, integration, and segregation of multiple talking faces on the basis of AV temporal synchrony even if the faces are not in their canonical orientation. In addition, we found that the magnitude of the preference for the target talking face was similar to that found in Experiment 1 in the Lewkowicz et al. [1] study. This finding is consistent with our prediction that A and V speech stream dynamics and their temporal congruence are likely to dominate responsiveness and that the configural talking-face template either plays a relatively minor role in responsiveness or no role at all.

The eye and mouth AOI data provided two interesting insights into the mechanisms that drove the multisensory binding, integration, and segregation of the multiple talking faces. First, it is clear from the far greater attention to the mouth than eyes that selective attention was driven by AV speech processing. This is in line with findings from previous studies showing that the relative degree of attention allocated to the eyes versus the mouth depends on whether a task requires processing of socially relevant cues or speech cues. When a task requires processing of socially relevant information, adults attend more to the eyes, but when a task requires processing of speech information, adults attend more to the mouth [21,35,38,67]. Of course, what makes the current findings especially interesting is that participants engaged in AV speech processing despite the fact that the mouth was inverted. The second insight provided by the eye and mouth data is that task difficulty affected the search strategy related to AV speech processing. When an audiovisually congruent talking face was present in the stimulus array, participants focused more attention on the mouth of the target talking face than the mouth of the distractor talking faces. This suggests that the audiovisually synchronized talking face popped out, making it easier to detect it. When, however, an audiovisually synchronized talking face was not present, participants had to deploy equal attention to the mouth of all four talking faces to find the talking face and thus comply with task demands.

Finally, the pupil size data did not provide unambiguous evidence that cognitive effort differed across the synchrony conditions. This is not surprising given that the four talking faces were the same, that they spoke the same V speech utterance, that the A utterance was the same as the V utterance, and that configural face processing was rendered ineffective due to inversion. As a result, participants did not have to process any other perceptual features except AV temporal synchrony. This, together with the pop-out effect, rendered the detection of the presence vs. absence of the target talking face relatively effortless.

## Experiment 2

The findings from Experiment 1 in the current study and those from Experiments 1 and 2 in the Lewkowicz et al. [1] study demonstrate unequivocally that AV temporal synchrony plays a powerful role in the segregation of cluttered multisensory scenes composed of multiple talking faces. Of course, social communication usually involves different individuals producing different linguistic utterances. As a result, perceivers usually have access to AV temporal synchrony cues as well as other perceptual cues during their interactions with other interlocutors. These additional perceptual cues are likely to facilitate multisensory binding, integration, and perceptual segregation. Indeed, as indicated earlier, Lewkowicz et al. [1] found that multisensory segregation of multi-talker scenes benefits from facial and vocal identity cues and that this is the case even when these cues are not temporally synchronized. As a result, in the current experiment, we asked whether the distinct linguistic utterances that are usually produced by different talkers also might contribute to multisensory segregation of multi-talker scenes.

To answer our question, we presented four different talking faces during each test trial, with each face now seen producing a distinct V speech utterance together with one of the four corresponding A speech utterances. The A utterance was temporally synchronized with its corresponding V utterance in the synchrony condition but desynchronized with it during the asynchrony condition. Crucially, however, the A utterance also was congruent with the corresponding V speech utterance in terms of facial, vocal, and linguistic identity cues in both synchrony conditions. In other words, the identity cues provided AV congruence information regardless of whether the V and A speech utterances were temporally synchronized or not. This made it possible to determine whether facial, vocal, and AV linguistic identity cues contribute to multisensory segregation of multi-talker scenes over-and-above AV temporal synchrony cues.

Given the Lewkowicz et al. [1] finding that facial and vocal identity cues facilitate multisensory integration and segregation, we predicted that linguistic identity cues also are likely to be beneficial. Thus, we not only expected to find a preference for the audiovisually synchronized talking face in the synchrony condition but also a preference for the talking face in the asynchrony condition whose facial, vocal, and AV linguistic identity cues corresponded and/or were congruent. Moreover, we expected that the preference for the audiovisually desynchronized talking face in the asynchrony condition would be greater in the current experiment than in Experiment 2 in the Lewkowicz et al. [1] study based on the assumption that linguistic identity cues play an independent role in multisensory binding, integration, and segregation of multiple talking faces.

### Method

#### Participants.

We tested 31 adults (15 females) who ranged between 19.1 and 37 years of age (mean age = 20.9 years, SD = 3.33 years). The recruitment for this experiment started on 4/4/2022 and ended on 10/7/2022. All procedures involving human participants were approved by the Yale University Institutional Review Board (Protocol #2000026034). All participants were English-speaking volunteers who gave their written informed consent prior to taking part in the study.

### Apparatus and Stimuli

The apparatus used in this experiment was the same as that used in Experiment 1. The composite videos presented during the practice and test trials were composed of different sets of female actors. During each test trial, we presented different combinations of four different actors (see Fig 1) drawn from a set of six actors. Each actor spoke a different utterance.

The experiment consisted of 16 synchrony and 16 asynchrony trials. During a given synchrony trial, one of the four A speech utterances was temporally synchronized with one of the four V utterances (the target) but desynchronized from the other three V utterances. In addition, this A utterance was congruent with the target talking face in terms of facial, vocal, and linguistic identity cues but incongruent with the other three V utterances (i.e., the distractors). During a given asynchrony trial, the A utterance was temporally desynchronized from all four V utterances, but it was still congruent with one of the talking faces in terms of facial, vocal, and linguistic identity cues. This talking face was designated the “virtual” target face because it was presented in the same quadrant as its counterpart in the synchrony condition trials. To desynchronize the corresponding A and V speech utterances in the asynchrony trials, the V speech stream was advanced by 2 s with respect to the A speech stream.

To create the composite videos for this experiment, first we filmed six different White, Caucasian women while they spoke two different utterances at a similar tempo. The pairs of utterances spoken by each actor were chosen randomly from a set of 7 different utterances. As a result, some of the utterances were spoken by more than one actor with the constraint that each experimental group (see below) was administered videos of 4 different actors speaking 4 different utterances, respectively. In all, we made 12 unique videos which represented 12 unique actor/utterance combinations. We then divided these 12 videos into 3 groups of four videos each and used these to construct a set of 32 composite videos for each of the 3 experimental groups. Sixteen of these composite videos comprised the synchrony condition while the other 16 composite videos comprised the asynchrony condition. To fully counterbalance the quadrant of target presentation within each experimental group, each of the four actors in each group appeared equally often in each of the 4 quadrants. As in Experiment 1, all four actors began talking simultaneously at the start of each test trial.

### Procedure

The procedure in this experiment was the same as that in Experiment 1. The test trials were presented in different random orders across three test groups and participants were randomly assigned to one of these three groups.

## Results

### Face AOIs

**Fig 5:**
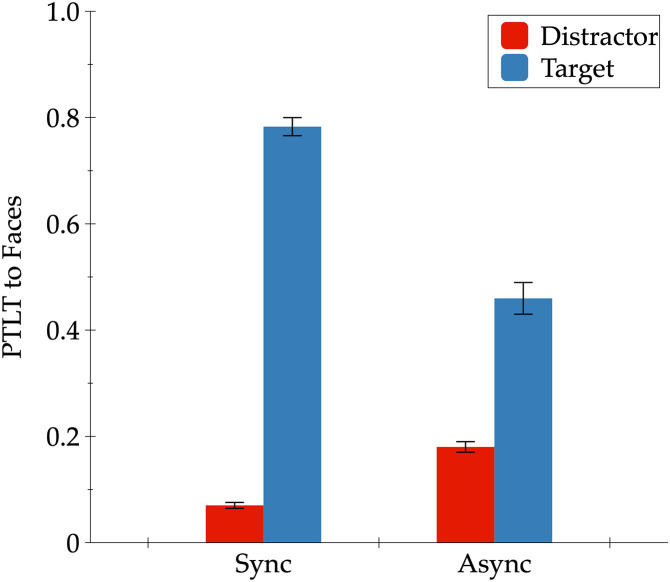
Selective attention to the temporally desynchronized (distractor) and synchronized (target) talking faces in the synchrony and asynchrony conditions in Experiment 2. Mean proportion of total looking time (PTLT) at the distractor and target faces across the two synchrony conditions. Error bars represent standard errors of the mean. The data underlying this figure can be found at https://osf.io/swzat/overview?view_only=e571661179694ec4a8cf1a8817af6b64.

Using LMM, we modeled the effects of Synchrony Condition and Stimulus Type on the PTLT directed at the face AOIs, with Synchrony Condition (2, Synchrony, Asynchrony) and Stimulus Type (2, Target, Distractor) as fixed effects and Subjects as random components. The main effects of Synchrony Condition and Stimulus Type were significant (*F*(1, 90) = 32.38, *p* < .001; *F*(1, 90) = 699.37, *p* < .001, respectively) as was their interaction, *F*(1, 90) = 130.38, *p* < .001. Fig 5 shows the two-way interaction and, as can be seen, participants not only exhibited a marked preference for the target talking face in the synchrony condition but they also exhibited a substantial preference for the virtual target talking face in the asynchrony condition.

Simple effects analyses of the Synchrony Condition x Stimulus Type interaction showed that the main effect of Synchrony Condition was due to greater looking at the target talking face than the distractor talking faces in the synchrony condition, *F*(1, 90) = 713.19, *p* < .001, as well as in the asynchrony condition, *F*(1, 90) = 113.49, *p* < .001. Additional simple effects analyses indicated that the 2-way interaction was due to greater looking at the target talking face in the synchrony condition than at the virtual target talking face in the asynchrony condition, *F*(1, 90) = 147.09, *p <* .001, and greater looking at the distractor talking faces in the asynchrony condition than at the distractor talking faces in the synchrony condition, *F*(1, 90) = 16.32, *p <* .001.

### Eye and Mouth AOIs

**Fig 6:**
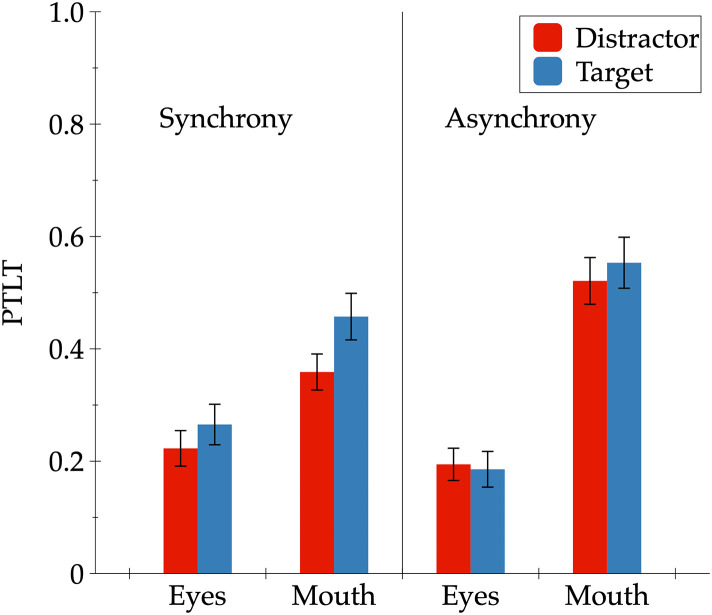
Selective attention to the eyes and mouth in Experiment 2. Mean proportion of total looking time to the eyes and mouth of the distractors and the target, respectively, in the two synchrony conditions in Experiment 2. Error bars represent standard errors of the mean. The data underlying this figure can be found at https://osf.io/swzat/overview?view_only=e571661179694ec4a8cf1a8817af6b64.

To examine selective attention to the eyes and mouth, we used LMM to model the effects of Synchrony Condition (2, Synchrony, Asynchrony), Stimulus Type (2, Distractor, Target), and AOI (2, eyes, mouth) as the fixed factors and the effects of Subjects as random components on the PTLT scores. The main effect of AOI was significant, *F*(1, 182) = 88.14, *p <* .001, as was the effect of an AOI x Synchrony Condition interaction, *F*(1, 182) = 10.73, *p =* .001. In contrast to Experiment 1, however, the AOI x Synchrony Condition x Stimulus interaction was not significant, *F*(1, 182) = 0.00, *p =* .962. Despite the absence of the triple interaction, the PTLT scores are plotted as a function of the three factors in Fig 6 to permit a direct comparison of eye and mouth looking across the two Experiments. As Fig 6 shows, and as attested by the main effect of AOI, overall attention to the mouth was greater than attention to the eyes. As also attested by the significant AOI x Synchrony Condition interaction, the main effect of AOI depended on the specific AOI in that looking at the mouth appeared to be greater in the asynchrony than in the synchrony condition whereas looking at the eyes appeared to be similar across the synchrony conditions. A simple effects analysis confirmed these differences in showing that looking at the mouth in the asynchrony condition was statistically greater than in the synchrony condition, *F*(1, 182) = 9.71, *p* = .002, but that looking at the eyes did not differ across the two synchrony conditions, *F*(1, 182) = 2.30, *p* = .131.

### Cognitive Effort

The findings from the Lewkowicz et al. [1] study and those from the current study clearly show that AV temporal synchrony cues make it relatively easy for adults to detect multisensory congruence in the synchrony condition. As noted earlier, this is most likely due to a pop-out effect of the target talking face. If that is the case, then the detection of multisensory congruence in the asynchrony condition should be more difficult given that AV temporal synchrony cues are absent in that condition. Despite this, Lewkowicz et al. [1] have shown that facial and vocal identity cues can facilitate the detection of AV congruence and there is little doubt that linguistic identity cues are also likely to facilitate it. Of course, this type of facilitation requires that participants learn the unique cue combinations that represent each individual talker. To do so, they must be able to [1] extract the equivalent A and V prosodic, phonetic, and lexical perceptual attributes that specify each unique talker’s AV utterances, [2] the unique, modality-specific, attributes that specify each face and its vocalizations, and [3] the V and A perceptual attributes of each talker’s semantically unique expressions. Engaging these various perceptual processes is certain to present a greater cognitive challenge than simply detecting whether one of the V speech streams is temporally synchronized with the A speech stream. Consequently, the detection of AV congruence in the asynchrony condition is likely to require greater cognitive effort and this should be reflected in larger pupil size than in the synchrony condition.

**Fig 7:**
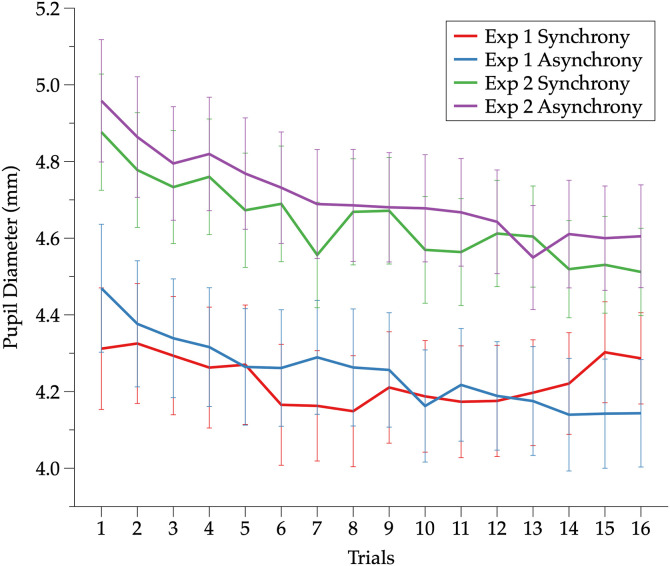
Cognitive effort. Average pupil diameter as a function of trials in the synchrony and asynchrony conditions in Experiment 2 in Experiment 1 for comparison. Error bars represent standard errors of the mean. The data underlying this figure can be found at https://osf.io/swzat/overview?view_only=e571661179694ec4a8cf1a8817af6b64.

To test this prediction in LMM, we modeled the effects of trial and synchrony condition on average pupil diameter in Experiment 2, with Trial [16] and Synchrony Condition (2, Synchrony, Asynchrony) as fixed effects and Subjects as random components. Fig 7 depicts the pupil diameter data from Experiment 2 and, for comparison purposes, also from Experiment 1. As can be seen, like in Experiment 1, pupil diameter decreased as a function of trial in Experiment 2, *F*(15, 1054) = 18.50, *p* < .001. This effect is most likely due to a combination of fatigue and cognitive disengagement in both experiments. Crucially, and in contrast to Experiment 1, pupil diameter in Experiment 2 was larger in the asynchrony than synchrony condition, *F*(1, 1054) = 28.11, *p* < .001. suggesting that participants exerted greater cognitive effort in the asynchrony condition as they attempted to find the missing target talking face.

In addition to the fact that pupil diameter was larger in the asynchrony than synchrony condition in Experiment 2, Fig 7 clearly shows that the overall magnitude of pupil diameter also was greater in Experiment 2 than in Experiment 1. An LMM analysis, with Synchrony Condition [2], Trial [16], and Experiment [2] as fixed factors and Subjects as random components did, indeed, confirm that this difference was statistically significant. Specifically, the LMM analysis yielded a main effect of Experiment, *F*(1, 65) = 4.84, *p* = .031, Synchrony Condition, *F*(1, 2015) = 20.04 *p* < .001, and Trial, *F*(15, 2015) = 19.54, *p* < .001, 2-way interactions of Experiment x Synchrony Condition, *F*(1, 2015) = 5.76, *p* = .016, Experiment x Trial, *F*(15, 2015) = 2.05, *p =* .01, Synchrony Condition x Trial, *F*(15, 2015) = 1.80, *p* = .03, and a 3-way interaction of Experiment x Synchrony Condition x Trial, *F*(15, 2015) = 1.91, *p* = .018.

The main effect of Experiment is due to a larger pupil in Experiment 2 than in Experiment 1 and is the most direct evidence that, overall, cognitive effort was greater in Experiment 2 while the main effect of Synchrony Condition reflects the fact that, overall, pupil diameter was larger in the asynchrony than synchrony condition. The main effect of Trial reflects decreasing pupil diameter as the experiment progressed and the Experiment x Synchrony Condition x Trial interaction shows that pupil dilation differed across trials in the two synchrony conditions across the two experiments. Together, these results indicate that the absence of an audiovisually congruent talking face in the stimulus array elicited greater cognitive effort than did its presence in Experiment 2 but that this was not the case in Experiment 1. Crucially, it should be noted that the statistical differences in pupil diameter across the two experiments cannot be attributed to differences in stimulus luminance across them. First, participants were tested with the same equipment in both experiments. Second, the stimulus materials for both experiments were constructed with the same settings in Premiere Pro. Finally, and most importantly, the brightness level of the screen during stimulus presentation in each experiment was identical (32 lux; measured at the same distance and at the position of a participant’s eyes from the screen). Therefore, the most reasonable conclusion from the pupil diameter data is that the greater stimulus processing demands in Experiment 2 were the most likely reason why participants expended greater cognitive effort in this experiment than in Experiment 1.

## Discussion

Like in Experiment 1, we found that participants exhibited a marked preference for the audiovisually synchronized talking face in the synchrony condition, greater attention to the mouth than eyes, and greater attention to the mouth in the asynchrony than synchrony condition. Interestingly, and in contrast to Experiment 1, we also found a substantial preference for the virtual target in the asynchrony condition. Finally, as expected, we found evidence of greater cognitive effort in the asynchrony than synchrony condition.

The preference for the audiovisually synchronized talking face in the synchrony condition confirms once again that AV temporal synchrony cues play a major role in the binding, integration, and perceptual segregation of multiple talking faces. The preference for the virtual target in the asynchrony condition demonstrates that person-specific and, therefore, unique facial, vocal, and AV linguistic identity cues that normally specify different talkers also contribute substantially to multisensory binding, integration, and segregation of multiple talking faces. The preference for the virtual target indicates that adults can take advantage of the various identity cues that are usually associated with different talkers to match an A speech stream with a corresponding talking face. In other words, it appears that adults can rely on some combination of the prosodic, phonetic, lexical, syntactic, and/or semantic attributes of an A speech stream to match it with some combination of the amodal and modality-specific visual attributes of a V speech stream produced by a particular talking face.

The findings of greater attention to the mouth, and of greater attention to the mouth in the asynchrony condition, replicate the results from Experiment 1. Like in Experiment 1, they provide evidence that the presence or absence of an audiovisually synchronized talking face in a stimulus array consisting of multiple talking faces affects the relative distribution of selective attention to the eyes and mouth and suggest that this is most likely due to active AV speech processing.

Finally, the pupil dilation findings in Experiment 2 are in line with our predictions. They demonstrate that the search for a missing audiovisually synchronized talking face requires more cognitive effort than does a search for such a face when it is present in the stimulus array. At first blush, this finding might seem at odds with the reasonable assumption that detection of the target talking face should be easier when more AV congruence cues are available. On closer examination, however, there is little doubt that the need to rapidly process multiple multisensory congruence cues, as opposed to one, requires more time and effort to determine which of the multiple talking faces is the audiovisually congruent one. This is especially the case when the only difference between the target and the virtual target talking face is that one is audiovisually synchronized while the other is not. The target talking face pops out and, thus, requires relatively little cognitive effort to detect it. By contrast, the virtual target talking face does not pop out and, as a result, participants must process the various identity cues to determine whether it is the audiovisually congruent one. In other words, detection of a stimulus that is highly salient and distinct from the other stimuli because it pops out requires less cognitive effort than the detection of a stimulus that is less salient and less distinct because it does not pop out.

### Cross-Study Comparisons

As noted earlier, the experiments in this study are a direct extension of the two experiments conducted by Lewkowicz et al. [1] using the same experimental paradigm. This afforded us the opportunity to compare the two sets of findings directly to see whether the specific experimental manipulations tested in this study, namely face inversion in Experiment 1 and the addition of linguistic identity cues in Experiment 2, affected responsiveness.

A preliminary visual inspection of the data from the two studies revealed one set of highly similar findings and two sets of different findings. The set of similar findings is that the audiovisually synchronized target talking face elicited essentially equal amounts of selective attention in Experiment 1 in each study regardless of whether the talking faces were upright or inverted (the implications of this finding are discussed in the General Discussion). One potentially informative set of different findings across the two studies is the pattern of eye and mouth preferences in Experiment 1 while the other is the pattern of face preferences in Experiment 2. To determine whether the different findings were statistically significant, we compared each, in turn, with LMMs and report the results of each analysis below.

### Eye and Mouth AOIs in Experiment 1 vs. Experiment 1 in Lewkowicz et al. [1]

**Fig 8:**
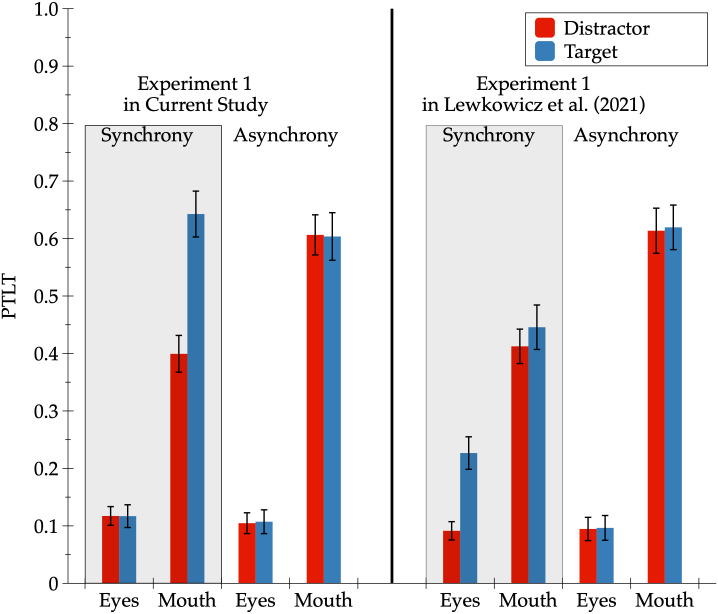
Comparison of selective attention to the eyes and mouth in Experiment 1 in the current study versus Experiment 1 in the Lewkowicz et al. [1] study. Mean proportion of total looking time to the eyes and mouth of the distractors and the target, respectively, in the synchrony and asynchrony conditions across the two experiments. Error bars represent standard errors of the mean. The data underlying this figure can be found at https://osf.io/swzat/overview?view_only=e571661179694ec4a8cf1a8817af6b64.

Inspection of the eye and mouth preferences across the two experiments indicated that looking at the eyes and mouth was remarkably similar as a function of stimulus in the asynchrony condition (see the unshaded portion of Fig 8) but that it differed as a function of stimulus in the synchrony condition (see the shaded areas of Fig 8). A closer examination of the preferences in the synchrony condition reveals that when participants viewed inverted talking faces in Experiment 1 in the current study, they looked equally at the eyes of the target and distractors but more at the mouth of the target than the mouth of the distractors. In contrast, when participants viewed upright talking faces in Experiment 1 in the Lewkowicz et al. [1] study, they looked longer at the eyes of the target than the distractors but they looked equally at the mouth of the target and the distractors.

Using LMM, we compared the eye and mouth AOI data from the synchrony condition in the two experiments, with Experiment [2], AOI [2], and Stimulus Type [2] as fixed factors and Subjects as random components. Results yielded main effects of AOI, *F*(1, 195) = 232.13, *p* < .001, and Stimulus Type, *F*(1, 195) = 18.93, *p* < .001, a 2-way interaction of Experiment x AOI, *F*(1, 195) = 13.49, *p* < .001, and a 3-way interaction of Experiment x AOI x Stimulus Type, *F*(1, 195) = 14.53, *p* < .001.

The Experiment x AOI x Stimulus Type interaction in the synchrony condition confirms that selective attention to the eyes and mouth of the distractors and the target differed across the two experiments. To identify the source of these differences, we conducted simple effects tests and found that, in Experiment 1 in the current study, looking at the eyes of the distractors and target did not differ, *F*(1, 195) = 0.03, *p* = .874, but that looking at the mouth of the target was greater than looking at the mouth of the distractors, *F*(1, 195) = 23.22, *p* < .001. Additionally, we found that, in Experiment 1 in the Lewkowicz et al. [1] study, looking at the eyes of the target was greater than looking at the eyes of the distractors, *F*(1, 195) = 10.42, *p* = .001, but that looking at the mouth of the distractors and the target did not differ, *F*(1, 195) = 0.63, *p* = .428.

Overall, these comparisons suggest that the greater face processing challenge posed by face inversion rendered the detection of AV temporal synchrony more difficult, requiring greater attention to the source of that information to ensure detection of the target talking face. Conversely, greater attention to the eyes in Experiment 1 in Lewkowicz et al. [1] indicates that participants were freer to explore the eyes given that they did not need to expend as much effort in determining which was the target talking face.

### Face AOIs in Experiment 2 vs. Experiment 2 in Lewkowicz et al. [1]

In their Experiment 2, Lewkowicz et al. [1] found that facial and vocal identity cues facilitated perceptual segregation in the absence of temporal synchrony cues. That is, participants preferred the virtual target over the distractors even though the A and V speech streams were not temporally synchronized. Given that Experiment 2 in the current study provided distinct linguistic cues in addition to facial and vocal identity cues, the question was whether the linguistic cues facilitated perceptual segregation independently of facial and vocal cues. To determine if this was the case, we compared the face preference data from Experiment 2 in the current study with the face preference data from Experiment 2 in the Lewkowicz et al. [1] study. Using LMM, we compared the face-AOI PTLT scores from the two experiments, with Stimulus Type [2], Synchrony Condition [2], and Experiment [2] as fixed factors and Subjects as random components. Results yielded main effects of Stimulus Type, *F*(1, 168) = 1271.32, *p* < .001, Synchrony Condition, *F*(1, 168) = 106.70, *p* < .001, and Experiment, *F*(1, 56) = 9.97, *p* = .003, 2-way interactions of Stimulus Type x Synchrony Condition, *F*(1, 168) = 434.26, *p* < .001, Stimulus Type x Experiment, *F*(1, 168) = 18.23, *p* < .001, and a marginal Synchrony Condition x Experiment, *F*(1, 168) = 3.12, *p* = .079, interaction, and a 3-way interaction of Stimulus Type x Synchrony Condition x Experiment, *F*(1, 168) = 13.20, *p* < .001.

**Fig 9:**
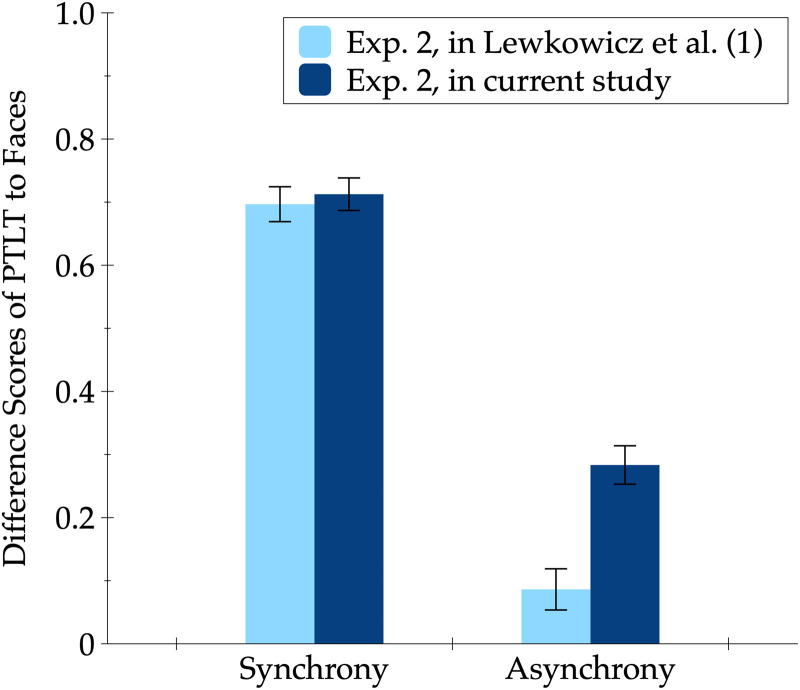
Comparison of selective attention to talking faces in Experiment 2 in the current study and Experiment 2 in Lewkowicz et al. [1]. The comparison is represented by PTLT difference scores computed by subtracting the distractor PTLT scores from the target PTLT scores. Error bars represent standard errors of the mean. The data underlying this figure can be found at https://osf.io/swzat/overview?view_only=e571661179694ec4a8cf1a8817af6b64.

The Stimulus Type x Synchrony Condition x Experiment interaction indicates that the preferences for each type of stimulus differed as a function of whether an audiovisually synchronized target stimulus was present or absent in the stimulus array and whether linguistic identity cues were present or not. To help visualize the source of this differential pattern of responsiveness, we calculated difference scores (i.e., target PTLT minus distractor PTLT) for the two synchrony conditions for each respective experiment and plotted them in Fig 9. The significant Synchrony Condition x Stimulus Type x Experiment interaction effect confirms that the relative magnitude of selective attention directed at the target vs. the distractors differed across the experiments. As Fig 9 shows, the source of this effect was the asynchrony condition where the magnitude of the difference in selective attention directed at the virtual target vs. the distractors was greater in the current study than in the Lewkowicz et al. [1] study. Indeed, simple effects tests showed that the magnitude of the preference for the target was the same in the synchrony condition in both experiments, *F*(1, 56) = 0.15, *p* = .703, but that the magnitude of the preference for the virtual target was greater in Experiment 2 in the current study than in Experiment 2 in the Lewkowicz et al. [1] study, *F*(1, 56) = 22.69, *p* < .001.

This comparison provides direct empirical proof that AV linguistic identity cues facilitate segregation of multiple talking faces and that they do so independently of facial and vocal identity cues. Moreover, these results suggest that the marked preference for an audiovisually synchronized talking face – when that face is part of a scene consisting of different talking faces producing distinct utterances – reflects two distinct processes in addition to the one involved in the detection of AV temporal synchrony. One is the formation of arbitrary associations between specific faces and their corresponding voices due to their co-occurrence while the second is the formation of common multisensory representations of the V and A speech streams specified by concurrent and congruent prosodic, phonetic, lexical, syntactic, and semantic multisensory attributes.

### General Discussion

The current study investigated multisensory binding, integration, and perceptual segregation of multiple talking faces with the same experimental paradigm employed in Lewkowicz et al.’s [1,2] studies of the MCPP in adults and in children. The current study extended the Lewkowicz et al. [1] study in adults by investigating three questions. First, in Experiment 1 we asked whether the canonical configural talking-face template is necessary for the perceptual segregation of multiple talking faces on the basis of AV temporal synchrony. Second, in Experiment 2 we asked whether the AV linguistic cues, which are usually associated with different talkers, contribute to perceptual segregation of multiple talkers. Finally, we asked whether specific task demands as well as the number and types of multisensory congruence cues that must be processed to solve the MCPP require different levels of cognitive effort.

To investigate the possible role of the canonical talking-face template in perceptual segregation of multiple talking faces, in Experiment 1 participants viewed four identical, inverted talking faces, speaking temporally jittered versions of the same utterance. While they watched the talking faces, they heard an A version of the same speech utterance which was either temporally synchronized with one of the talking faces during half the trials or desynchronized from all of them in the other half of the trials. To investigate whether AV linguistic cues contribute to the perceptual segregation of multiple talking faces, in Experiment 2 participants viewed four distinct upright talking faces speaking different speech utterances and heard one of the four corresponding A utterances. Like in Experiment 1, the A utterance was either temporally synchronized with one of the talking faces during half the trials or desynchronized from all of them in the other half of the trials. Crucially, in this experiment, the A utterance corresponded to one of the talking faces regardless of whether it was temporally synchronized with it or not because it also corresponded to that talking face in terms of its unique facial, vocal, and linguistic attributes. Finally, to investigate whether different task demands and differential stimulus processing demands elicit different levels of cognitive effort, we examined pupillary response in both experiments.

Measures of selective attention to the four talking faces in the two experiments revealed a marked preference for the temporally synchronized talking face regardless of whether the four talking faces were inverted or upright. This preference replicates similar findings in the Lewkowicz et al. [1] study and reinforces the conclusion that the temporal synchrony that normally links the dynamics of corresponding A and V speech streams plays a powerful role in their binding and integration into a unitary AV event and that this, in turn, facilitates the perceptual segregation of competing talking faces. The results from Experiment 1 are particularly interesting because they add new evidence in support of the claim that AV temporal synchrony cues not only provide a powerful basis for solving the MCPP but that they dominate responsiveness to other perceptual features of talking faces. In the present case, this new evidence demonstrates that temporal AV synchrony cues are a more salient perceptual feature of talking faces than is their spatial orientation. At first blush, this might seem to be surprising given that the developmentally acquired talking-face template for AV speech is such an intrinsic feature of our everyday perceptual experience with talking faces. On further examination, however, this may not be surprising in the context of the MCPP where binding, integration, and perceptual segregation of the bound and integrated entities (i.e., the AV speech streams of different talkers) is the primary task facing a perceiver.

The one possible caveat to the interpretation of the data from Experiment 1 is that all four talking faces in this experiment were identical and, thus, that the perceptual segregation of the multiple talking faces did not have to rely on face recognition processes *per se*. In other words, even though face inversion disrupts holistic face processing, there was little need for face processing in this experiment because the faces were identical and, as a result, participants were free to allocate all their attention to the dynamic AV information. This interpretation leads to the possibility that face inversion may have more disruptive effects on the segregation of multiple talking faces when distinct holistic facial features are available. Indeed, and consistent with this possibility, Experiment 2 in the Lewkowicz et al. [1] study indicated that when facial identity cues are available in the stimulus array, participants take advantage of them to perceptually segregate multiple talking faces. Given this, it may be that face inversion might interfere with the beneficial effects of facial identity cues on the binding, integration, and perceptual segregation of multiple talking faces. Currently, this is an open question. In the meantime, however, it is interesting to note that the present results are consistent with findings from other studies indicating that, at least in the case of the processing of consonant-vowel syllables, face inversion has minimal disruptive effects [18,43,44,71].

Even though the perceptual salience of the talking-face template was dominated by the greater perceptual salience of AV temporal synchrony cues, we obtained evidence that face inversion affected the relative distribution of selective attention to the eyes and mouth in Experiment 1 in the current study and in the earlier Lewkowicz et al. [1] study. This evidence showed that the relative proportion of selective attention directed to the mouth vs. the eyes was greater when the faces were inverted than when the faces were upright, indicating that inversion had a negative effect on multisensory processing.

The results from Experiment 2 indicated clearly that linguistic identity cues, on top of facial and vocal identity cues, facilitate perceptual segregation of multiple talking faces in the absence of AV temporal synchrony cues. In other words, the results from Experiment 2 indicated that linguistic identity cues can facilitate segregation independently of facial and vocal identity cues. This was evident in the fact that the magnitude of the preference for the virtual target in the asynchrony condition in the current Experiment 2 was statistically greater than in the asynchrony condition in Experiment 2 in the Lewkowicz et al. [1] study. The primary difference between Experiment 2 in the current study and Experiment 2 in the Lewkowicz et al. [1] study was that linguistic, facial, and vocal identity cues were available in the current study whereas only facial and vocal identity cues were available in the Lewkowicz et al. [1] study. Thus, the greater preference for the virtual target talking face in the current study in Experiment 2 can only be interpreted as reflecting the independent contribution of linguistic identity cues to the detection of multisensory congruence and, ultimately, to perceptual segregation.

The contribution of the various person-specific identity cues to the binding, integration, and segregation of multiple talking faces raises some interesting questions for future studies. For example, is one of these identity cues primary for adults or does each cue contribute equally? One possibility is that the relative functional importance of the various identity cues is hierarchically organized and that the hierarchy changes as a function of specific cognitive demands and their interaction with the ease of cue detection. Another interesting question is whether the relative functional importance of the various identity cues changes with development. For example, how likely is it that prosodic and phonetic identity cues are primary in early development and that they become gradually supplanted by lexical, syntactic, and semantic identity cues as children grow and acquire increasingly more sophisticated cognitive abilities?

### Mechanisms Underlying Multisensory Segregation of Multiple Talking Faces

Overall, the eye and mouth data from the current study replicate those from the Lewkowicz et al. [1] study. They indicate that face preferences were driven primarily by selective attention to the mouth and that this varied as a function of the presence or absence of an audiovisually synchronized talking face in the stimulus array or not. When it was absent, attention to the mouth was greater, suggesting that its absence compelled participants to attend more to the mouth to ensure that they did not miss AV speech congruence and, thus, that they complied with task demands. Crucially, it should be noted that the presentation of an audiovisually congruent talking face was unpredictable on any given trial because of trial randomization. Therefore, when an audiovisually congruent talking face was present on a given trial, it was likely that it popped out in a manner similar to the way that simple objects synchronized with a simple sound pop out from a scene consisting of multiple objects which are not synchronized with that sound [57,61]. In the case of fluent AV speech, such a pop-out effect renders the search for the target talking face easier and, thus, requires less attention to the mouth.

The greater focus on the mouth is clear evidence that AV speech processing drove responsiveness. Of course, this was required by the assigned experimental task; otherwise, it would have been impossible for the participants to identify the audiovisually congruent talking face. This conclusion is further bolstered by the fact that participants deployed more attention to the eyes and less attention to the mouth in Experiment 2 than in Experiment 1. This is consistent with findings that relative differences in selective attention to the eyes vs. the mouth are associated with the type of perceptual processing required in a task. That is, when speech processing is not required, adults tend to attend more to the eyes than mouth [21,67,72] but when speech processing is required, adults tend to attend more to the mouth [35,37,38,67]. In the present case, in Experiment 1 participants could only rely on AV speech processing to find the talking face. In contrast, in Experiment 2 participants could also rely on modality-specific identity cues and, as a result, could depend less on AV speech processing to find the talking face. Of course, it might be argued that face inversion in Experiment 1 rendered the detection of the target talking face more difficult because the processing of face identity information was disrupted. It should be noted, however, that the faces in Experiment 1 were identical and, thus, that facial identity information would not have been helpful even if the faces had been presented upright. Therefore, the greater attention to the mouth relative to the eyes in Experiment 1 suggests that a greater focus on the mouth was essential to detecting the talking face, especially because the V speech stream was now specified in a non-canonical way.

### Cognitive Effort

The pupil diameter data indicated that pupil size did not differ across the two synchrony conditions in Experiment 1 but that it was greater in the asynchrony than synchrony condition in Experiment 2. Together, these findings indicate that participants did not exert greater cognitive effort in their search for the audiovisually synchronized talking face when it was absent from the stimulus array in Experiment 1 but that they did in Experiment 2. Furthermore, the pupil diameter data showed that, regardless of synchrony condition, pupil diameter was greater in Experiment 2 than in Experiment 1. This shows that participants exerted more cognitive effort in Experiment 2 than in Experiment 1 to comply with task demands. The most likely reason for this finding is that participants had to process multiple and qualitatively different multisensory congruence cues in Experiment 2 and that this required them to engage different types of processing mechanisms. Specifically, when AV temporal synchrony cues accompanied facial, vocal, and AV linguistic identity cues, identification of the audiovisually congruent talking face was likely rendered relatively easy due to pop-out effects. When, however, AV temporal synchrony cues were absent, the processing of the multisensory congruence identity cues required greater effort because participants had to rely on working memory to recall previously associated features of corresponding A and V speech streams. Despite this, the greater cognitive effort expended in the detection of audiovisually congruent talking faces based solely on multisensory identity cues demonstrates how flexible our brains are in adapting to the challenges of the MCPP.

### Summary & Conclusion

The MCPP is a constant challenge during social communication. The current study investigated the mechanisms underlying the binding and integration of the auditory and visual attributes of multiple talkers and the ultimate perceptual segregation of the integrated multisensory representations of those talkers that perceivers must successfully accomplish to solve the MCPP. In Experiment 1 we found that temporal AV synchrony cues are a more salient perceptual feature of talking faces than their spatial orientation, even though the developmentally acquired talking-face template creates the perceptual expectation that talking faces are typically upright. In Experiment 2 we found that face, voice, and linguistic identity cues play an important role in the solution of the MCPP in that they provide an independent source of multisensory congruence information that enables adults to bind, integrate, and perceptually segregate multiple talking faces.

Overall, the present study demonstrates that we possess powerful multisensory processing mechanisms that enable us to rapidly and efficiently solve the MCPP. Doing so undoubtedly makes it possible for us to successfully engage in seemingly fluid and effortless social communication. Given this, is it possible that an inability to deal with the MCPP in a rapid and efficient manner might negatively affect social communication? This is certainly a possibility in special populations where sensory, perceptual, and/or cognitive processing mechanisms are not functioning properly due to some developmental anomaly such as, for example, autism spectrum disorder. The MCPP experimental paradigm offers a novel way to investigate such questions because it places an especially heavy processing burden on the perceptual system. A system that breaks down under such heavy processing demands often provides the best clues to what causes that system to malfunction and, thereby, offers clues to the best ways to ameliorate those malfunctions.

## Supporting information

S1 File Results of linear mixed-effects model analyses for Experiments 1 and 2, respectively, as well as of cross-study comparisons. (DOCX)
