# Supplementary material for: The multisensory cocktail party problem in adults: The effects of the configural talking-face template and of facial, vocal, and linguistic identity cues on its solution
Source: PLoS One. 2026 Jul 28;21(7):e0354673. doi: 10.1371/journal.pone.0354673 (PMC13411873; doi:10.1371/journal.pone.0354673)
Supplement: S1 File — (DOCX) [file pone.0354673.s001.docx]

**Supplementary File #1**

**“The multisensory cocktail party problem in adults: The effects of the configural talking-face template and of facial, vocal, and linguistic identity cues on its solution”**

**David J. Lewkowicz & Julia McClellan**

**Experiment 1**

**Face AOIs**

*LMM Analysis of PTLT Scores*

**Mixed Model**

| Model Info | | |
| --- | --- | --- |
| Info |  |  |
| Model Type | Mixed Model | Linear Mixed model for continuous y |
| Model | lme | PTLT ~ 1 + `Sync Condition` + Stimulus + `Sync Condition`:Stimulus + ( 1 \| Subject ) |
| Distribution | Gaussian | Normal distribution of residuals |
| Direction | y | Dependend variable scores |
| Residuals | Compound Symmetry | within cluster Subject |
| Sample size | 108 |  |
| Converged | yes |  |
| Y transform | none |  |
| C.I. method | Wald |  |

**Model Results**

| Model Fit | | | | |
| --- | --- | --- | --- | --- |
| Type | R² | df | LRT X² | p |
| Conditional | 0.95 | 5 | 314.26 | <.001 |
| Marginal | 0.95 | 3 | 278.61 | <.001 |

| Fixed Effects Omnibus Tests | | | | |
| --- | --- | --- | --- | --- |
|  | F | df | df (res) | p |
| Sync Condition | 218.83 | 1 | 78.00 | <.001 |
| Stimulus | 764.03 | 1 | 78.00 | <.001 |
| Sync Condition ✻ Stimulus | 876.80 | 1 | 78.00 | <.001 |

| Parameter Estimates (Fixed coefficients) | | | | | | | | |
| --- | --- | --- | --- | --- | --- | --- | --- | --- |
|  | | | | 95% Confidence Intervals | |  | | |
| Names | Effect | Estimate | SE | Lower | Upper | df | t | p |
| (Intercept) | (Intercept) | 0.34 | 0.00 | 0.33 | 0.35 | 78.00 | 88.49 | <.001 |
| Sync Condition1 | Async - Sync | -0.19 | 0.01 | -0.21 | -0.16 | 78.00 | -14.79 | <.001 |
| Stimulus1 | Target - Distractor | 0.35 | 0.01 | 0.33 | 0.38 | 78.00 | 27.64 | <.001 |
| Sync Condition1 ✻ Stimulus1 | (Async - Sync) ✻ (Target - Distractor) | -0.75 | 0.03 | -0.81 | -0.70 | 78.00 | -29.61 | <.001 |

| Random Components | | | | | |
| --- | --- | --- | --- | --- | --- |
| Groups | Name | Variance | SD | ICC | rho |
| Subject | (Intercept) | 0.00 | 0.00 | 0.00 | -0.19 |
| Residual |  | 0.00 | 0.06 |  |  |
| Note. Number of Obs: 108 , Number of groups: Subject 27 | | | | | |

**Experiment 1**

**Face AOIs**

*LMM Analysis of Raw Looking Time Scores*

**Mixed Model**

| Model Info | | |
| --- | --- | --- |
| Info |  |  |
| Model Type | Mixed Model | Linear Mixed model for continuous y |
| Model | lmer | LookTime ~ 1 + SyncCondition + Stimulus + SyncCondition:Stimulus + ( 1 \| Subject ) |
| Distribution | Gaussian | Normal distribution of residuals |
| Direction | y | Dependend variable scores |
| Optimizer | bobyqa |  |
| DF method | Satterthwaite |  |
| Sample size | 3456 |  |
| Converged | yes |  |
| Y transform | none |  |
| C.I. method | Wald |  |

**Model Results**

| Model Fit | | | | |
| --- | --- | --- | --- | --- |
| Type | R² | df | LRT X² | p |
| Conditional | 0.72 | 8 | 4273.08 | <.001 |
| Marginal | 0.68 | 7 | 4220.90 | <.001 |

| Fixed Effects Omnibus Tests | | | | |
| --- | --- | --- | --- | --- |
|  | F | df | df (res) | p |
| SyncCondition | 1.12 | 1 | 3422.00 | .290 |
| Stimulus | 1281.60 | 3 | 3422.00 | <.001 |
| SyncCondition ✻ Stimulus | 1483.52 | 3 | 3422.00 | <.001 |

| Parameter Estimates (Fixed coefficients) | | | | | | | | |
| --- | --- | --- | --- | --- | --- | --- | --- | --- |
|  | | | | 95% Confidence Intervals | |  | | |
| Names | Effect | Estimate | SE | Lower | Upper | df | t | p |
| (Intercept) | (Intercept) | 3211.37 | 127.84 | 2960.73 | 3462.02 | 26.00 | 25.12 | <.001 |
| SyncCondition1 | 2 - 1 | 67.93 | 64.13 | -57.80 | 193.66 | 3422.00 | 1.06 | .290 |
| Stimulus1 | 2 - 1 | 10.36 | 90.69 | -167.45 | 188.17 | 3422.00 | 0.11 | .909 |
| Stimulus2 | 3 - 1 | 11.37 | 90.69 | -166.44 | 189.18 | 3422.00 | 0.13 | .900 |
| Stimulus3 | 4 - 1 | 4598.65 | 90.69 | 4420.84 | 4776.46 | 3422.00 | 50.71 | <.001 |
| SyncCondition1 ✻ Stimulus1 | (2 - 1) ✻ (2 - 1) | 33.80 | 181.38 | -321.82 | 389.42 | 3422.00 | 0.19 | .852 |
| SyncCondition1 ✻ Stimulus2 | (2 - 1) ✻ (3 - 1) | 165.27 | 181.38 | -190.35 | 520.89 | 3422.00 | 0.91 | .362 |
| SyncCondition1 ✻ Stimulus3 | (2 - 1) ✻ (4 - 1) | -9812.41 | 181.38 | -10168.03 | -9456.79 | 3422.00 | -54.10 | <.001 |

| Random Components | | | | |
| --- | --- | --- | --- | --- |
| Groups | Name | Variance | SD | ICC |
| Subject | (Intercept) | 413488.21 | 643.03 | 0.10 |
| Residual |  | 3553003.68 | 1884.94 |  |
| Note. Number of Obs: 3456 , Number of groups: Subject 27 | | | | |

**Experiment 1**

**Eye and Mouth AOIs**

*LMM Analysis of PTLT Scores*

# **Mixed Model**

| Model Info | | |
| --- | --- | --- |
| Info |  |  |
| Model Type | Mixed Model | Linear Mixed model for continuous y |
| Model | lmer | PTLT ~ 1 + `Sync Condition` + AOI + Stimulus + `Sync Condition`:AOI + `Sync Condition`:Stimulus + AOI:Stimulus + `Sync Condition`:AOI:Stimulus + ( 1 \| Subject ) |
| Distribution | Gaussian | Normal distribution of residuals |
| Direction | y | Dependend variable scores |
| Optimizer | bobyqa |  |
| DF method | Satterthwaite |  |
| Sample size | 216 |  |
| Converged | yes |  |
| Y transform | none |  |
| C.I. method | Wald |  |

**Model Results**

| Model Fit | | | | |
| --- | --- | --- | --- | --- |
| Type | R² | df | LRT X² | p |
| Conditional | 0.72 | 8 | 276.01 | <.001 |
| Marginal | 0.71 | 7 | 276.01 | <.001 |

| Fixed Effects Omnibus Tests | | | | |
| --- | --- | --- | --- | --- |
|  | F | df | df (res) | p |
| Sync Condition | 3.07 | 1 | 182.00 | .081 |
| AOI | 501.26 | 1 | 182.00 | <.001 |
| Stimulus | 7.38 | 1 | 182.00 | .007 |
| Sync Condition ✻ AOI | 5.56 | 1 | 182.00 | .019 |
| Sync Condition ✻ Stimulus | 7.17 | 1 | 182.00 | .008 |
| AOI ✻ Stimulus | 8.46 | 1 | 182.00 | .004 |
| Sync Condition ✻ AOI ✻ Stimulus | 8.14 | 1 | 182.00 | .005 |

| Parameter Estimates (Fixed coefficients) | | | | | | | | |
| --- | --- | --- | --- | --- | --- | --- | --- | --- |
|  | | | | 95% Confidence Intervals | |  | | |
| Names | Effect | Estimate | SE | Lower | Upper | df | t | p |
| (Intercept) | (Intercept) | 0.34 | 0.01 | 0.32 | 0.37 | 26.00 | 30.52 | <.001 |
| Sync Condition1 | Async - Sync | 0.04 | 0.02 | -0.00 | 0.08 | 182.00 | 1.75 | .081 |
| AOI1 | Mouth - Eyes | 0.49 | 0.02 | 0.45 | 0.54 | 182.00 | 22.39 | <.001 |
| Stimulus1 | Target - Distractor | 0.06 | 0.02 | 0.02 | 0.10 | 182.00 | 2.72 | .007 |
| Sync Condition1 ✻ AOI1 | (Async - Sync) ✻ (Mouth - Eyes) | 0.10 | 0.04 | 0.02 | 0.19 | 182.00 | 2.36 | .019 |
| Sync Condition1 ✻ Stimulus1 | (Async - Sync) ✻ (Target - Distractor) | -0.12 | 0.04 | -0.20 | -0.03 | 182.00 | -2.68 | .008 |
| AOI1 ✻ Stimulus1 | (Mouth - Eyes) ✻ (Target - Distractor) | 0.13 | 0.04 | 0.04 | 0.22 | 182.00 | 2.91 | .004 |
| Sync Condition1 ✻ AOI1 ✻ Stimulus1 | (Async - Sync) ✻ (Mouth - Eyes) ✻ (Target - Distractor) | -0.25 | 0.09 | -0.43 | -0.08 | 182.00 | -2.85 | .005 |

| Random Components | | | | |
| --- | --- | --- | --- | --- |
| Groups | Name | Variance | SD | ICC |
| Subject | (Intercept) | 0.00 | 0.01 | 0.00 |
| Residual |  | 0.03 | 0.16 |  |
| Note. Number of Obs: 216 , Number of groups: Subject 27 | | | | |

**Experiment 1**

**Cognitive Effort**

*LMM Analysis of Pupil Diameter*

**Mixed Model**

| Model Info | | |
| --- | --- | --- |
| Info |  |  |
| Model Type | Mixed Model | Linear Mixed model for continuous y |
| Model | lme | `Pupil Diameter` ~ 1 + Trial + `Sync Condition` + Trial:`Sync Condition` + ( 1 \| Subject ) |
| Distribution | Gaussian | Normal distribution of residuals |
| Direction | y | Dependend variable scores |
| Residuals | Compound Symmetry | within cluster Subject |
| Sample size | 1024 |  |
| Converged | yes |  |
| Y transform | none |  |
| C.I. method | Wald |  |

**Model Results**

| Model Fit | | | | |
| --- | --- | --- | --- | --- |
| Type | R² | df | LRT X² | p |
| Conditional | 0.93 | 33 | 2443.13 | <.001 |
| Marginal | 0.01 | 31 | -26.10 | 1.000 |

| Fixed Effects Omnibus Tests | | | | |
| --- | --- | --- | --- | --- |
|  | F | df | df (res) | p |
| Trial | 5.10 | 15 | 961.00 | <.001 |
| Sync Condition | 1.82 | 1 | 961.00 | .177 |
| Trial ✻ Sync Condition | 2.48 | 15 | 961.00 | .001 |

| Parameter Estimates (Fixed coefficients) | | | | | | | | |
| --- | --- | --- | --- | --- | --- | --- | --- | --- |
|  | | | | 95% Confidence Intervals | |  | | |
| Names | Effect | Estimate | SE | Lower | Upper | df | t | p |
| (Intercept) | (Intercept) | 4.24 | 0.15 | 3.94 | 4.54 | 961.00 | 27.90 | <.001 |
| Trial1 | 2 - 1 | -0.04 | 0.04 | -0.12 | 0.04 | 961.00 | -0.98 | .329 |
| Trial2 | 3 - 1 | -0.07 | 0.04 | -0.15 | 0.01 | 961.00 | -1.83 | .067 |
| Trial3 | 4 - 1 | -0.10 | 0.04 | -0.18 | -0.02 | 961.00 | -2.50 | .013 |
| Trial4 | 5 - 1 | -0.12 | 0.04 | -0.20 | -0.04 | 961.00 | -3.04 | .002 |
| Trial5 | 6 - 1 | -0.18 | 0.04 | -0.26 | -0.10 | 961.00 | -4.36 | <.001 |
| Trial6 | 7 - 1 | -0.16 | 0.04 | -0.24 | -0.08 | 961.00 | -4.05 | <.001 |
| Trial7 | 8 - 1 | -0.18 | 0.04 | -0.26 | -0.11 | 961.00 | -4.55 | <.001 |
| Trial8 | 9 - 1 | -0.16 | 0.04 | -0.24 | -0.08 | 961.00 | -3.87 | <.001 |
| Trial9 | 10 - 1 | -0.22 | 0.04 | -0.30 | -0.14 | 961.00 | -5.31 | <.001 |
| Trial10 | 11 - 1 | -0.20 | 0.04 | -0.27 | -0.12 | 961.00 | -4.81 | <.001 |
| Trial11 | 12 - 1 | -0.21 | 0.04 | -0.29 | -0.13 | 961.00 | -5.13 | <.001 |
| Trial12 | 13 - 1 | -0.20 | 0.04 | -0.28 | -0.12 | 961.00 | -5.04 | <.001 |
| Trial13 | 14 - 1 | -0.21 | 0.04 | -0.29 | -0.13 | 961.00 | -5.18 | <.001 |
| Trial14 | 15 - 1 | -0.17 | 0.04 | -0.25 | -0.09 | 961.00 | -4.14 | <.001 |
| Trial15 | 16 - 1 | -0.18 | 0.04 | -0.26 | -0.10 | 961.00 | -4.33 | <.001 |
| Sync Condition1 | Async - Sync | 0.02 | 0.01 | -0.01 | 0.05 | 961.00 | 1.35 | .177 |
| Trial1 ✻ Sync Condition1 | (2 - 1) ✻ (Async - Sync) | -0.11 | 0.08 | -0.27 | 0.05 | 961.00 | -1.31 | .191 |
| Trial2 ✻ Sync Condition1 | (3 - 1) ✻ (Async - Sync) | -0.11 | 0.08 | -0.27 | 0.05 | 961.00 | -1.38 | .168 |
| Trial3 ✻ Sync Condition1 | (4 - 1) ✻ (Async - Sync) | -0.10 | 0.08 | -0.26 | 0.06 | 961.00 | -1.28 | .200 |
| Trial4 ✻ Sync Condition1 | (5 - 1) ✻ (Async - Sync) | -0.16 | 0.08 | -0.32 | -0.00 | 961.00 | -2.01 | .045 |
| Trial5 ✻ Sync Condition1 | (6 - 1) ✻ (Async - Sync) | -0.06 | 0.08 | -0.22 | 0.10 | 961.00 | -0.76 | .449 |
| Trial6 ✻ Sync Condition1 | (7 - 1) ✻ (Async - Sync) | -0.03 | 0.08 | -0.19 | 0.13 | 961.00 | -0.38 | .703 |
| Trial7 ✻ Sync Condition1 | (8 - 1) ✻ (Async - Sync) | -0.04 | 0.08 | -0.20 | 0.12 | 961.00 | -0.54 | .592 |
| Trial8 ✻ Sync Condition1 | (9 - 1) ✻ (Async - Sync) | -0.11 | 0.08 | -0.27 | 0.05 | 961.00 | -1.38 | .169 |
| Trial9 ✻ Sync Condition1 | (10 - 1) ✻ (Async - Sync) | -0.18 | 0.08 | -0.34 | -0.02 | 961.00 | -2.25 | .025 |
| Trial10 ✻ Sync Condition1 | (11 - 1) ✻ (Async - Sync) | -0.11 | 0.08 | -0.27 | 0.05 | 961.00 | -1.40 | .162 |
| Trial11 ✻ Sync Condition1 | (12 - 1) ✻ (Async - Sync) | -0.14 | 0.08 | -0.30 | 0.01 | 961.00 | -1.78 | .076 |
| Trial12 ✻ Sync Condition1 | (13 - 1) ✻ (Async - Sync) | -0.18 | 0.08 | -0.34 | -0.02 | 961.00 | -2.21 | .027 |
| Trial13 ✻ Sync Condition1 | (14 - 1) ✻ (Async - Sync) | -0.24 | 0.08 | -0.40 | -0.08 | 961.00 | -2.94 | .003 |
| Trial14 ✻ Sync Condition1 | (15 - 1) ✻ (Async - Sync) | -0.32 | 0.08 | -0.48 | -0.16 | 961.00 | -3.91 | <.001 |
| Trial15 ✻ Sync Condition1 | (16 - 1) ✻ (Async - Sync) | -0.30 | 0.08 | -0.46 | -0.14 | 961.00 | -3.70 | <.001 |

| Random Components | | | | | |
| --- | --- | --- | --- | --- | --- |
| Groups | Name | Variance | SD | ICC | rho |
| Subject | (Intercept) | 0.74 | 0.86 | 0.93 | 0.00 |
| Residual |  | 0.05 | 0.23 |  |  |
| Note. Number of Obs: 1024 , Number of groups: Subject 32 | | | | | |

**Experiment 2**

**Face AOIs**

*LMM Analysis of PTLT Scores*

**Mixed Model**

| Model Info | | |
| --- | --- | --- |
| Info |  |  |
| Model Type | Mixed Model | Linear Mixed model for continuous y |
| Model | lme | PTLT ~ 1 + `Sync Condition` + Stimulus + `Sync Condition`:Stimulus + ( 1 \| Subject ) |
| Distribution | Gaussian | Normal distribution of residuals |
| Direction | y | Dependend variable scores |
| Residuals | Compound Symmetry | within cluster Subject |
| Sample size | 124 |  |
| Converged | yes |  |
| Y transform | none |  |
| C.I. method | Wald |  |

**Model Results**

| Model Fit | | | | |
| --- | --- | --- | --- | --- |
| Type | R² | df | LRT X² | p |
| Conditional | 0.89 | 5 | 251.51 | <.001 |
| Marginal | 0.89 | 3 | 213.11 | <.001 |

| Fixed Effects Omnibus Tests | | | | |
| --- | --- | --- | --- | --- |
|  | F | df | df (res) | p |
| Sync Condition | 32.38 | 1 | 90.00 | <.001 |
| Stimulus | 699.37 | 1 | 90.00 | <.001 |
| Sync Condition ✻ Stimulus | 130.38 | 1 | 90.00 | <.001 |

| Parameter Estimates (Fixed coefficients) | | | | | | | | |
| --- | --- | --- | --- | --- | --- | --- | --- | --- |
|  | | | | 95% Confidence Intervals | |  | | |
| Names | Effect | Estimate | SE | Lower | Upper | df | t | p |
| (Intercept) | (Intercept) | 0.37 | 0.01 | 0.36 | 0.39 | 90.00 | 58.89 | <.001 |
| Sync Condition1 | { 1*Sync, -1*Async } | 0.05 | 0.01 | 0.03 | 0.07 | 90.00 | 5.69 | <.001 |
| Stimulus1 | { 1*Distractor, -1*Target } | -0.25 | 0.01 | -0.27 | -0.23 | 90.00 | -26.45 | <.001 |
| Sync Condition1 ✻ Stimulus1 | ({ 1*Sync, -1*Async }) ✻ ({ 1*Distractor, -1*Target }) | -0.11 | 0.01 | -0.13 | -0.09 | 90.00 | -11.42 | <.001 |

| Random Components | | | | | |
| --- | --- | --- | --- | --- | --- |
| Groups | Name | Variance | SD | ICC | rho |
| Subject | (Intercept) | 0.00 | 0.01 | 0.01 | -0.17 |
| Residual |  | 0.01 | 0.10 |  |  |
| Note. Number of Obs: 124 , Number of groups: Subject 31 | | | | | |

**Experiment 2**

**Eye and Mouth AOIs**

*LMM Analysis of PTLT Scores*

# **Mixed Model**

| Model Info | | |
| --- | --- | --- |
| Info |  |  |
| Model Type | Mixed Model | Linear Mixed model for continuous y |
| Model | lme | PTLT ~ 1 + AOI + `Sync Condition` + Stimulus + AOI:`Sync Condition` + AOI:Stimulus + `Sync Condition`:Stimulus + AOI:`Sync Condition`:Stimulus + ( 1 \| Subject ) |
| Distribution | Gaussian | Normal distribution of residuals |
| Direction | y | Dependend variable scores |
| Residuals | Compound Symmetry | within cluster Subject |
| Sample size | 216 |  |
| Converged | yes |  |
| Y transform | none |  |
| C.I. method | Wald |  |

## **Model Results**

| Model Fit | | | | |
| --- | --- | --- | --- | --- |
| Type | R² | df | LRT X² | p |
| Conditional | 0.35 | 9 | 64.20 | <.001 |
| Marginal | 0.34 | 7 | 55.45 | <.001 |

| Fixed Effects Omnibus Tests | | | | |
| --- | --- | --- | --- | --- |
|  | F | df | df (res) | p |
| AOI | 88.14 | 1 | 182.00 | <.001 |
| Sync Condition | 1.28 | 1 | 182.00 | .259 |
| Stimulus | 2.22 | 1 | 182.00 | .138 |
| AOI ✻ Sync Condition | 10.73 | 1 | 182.00 | .001 |
| AOI ✻ Stimulus | 0.78 | 1 | 182.00 | .379 |
| Sync Condition ✻ Stimulus | 1.10 | 1 | 182.00 | .296 |
| AOI ✻ Sync Condition ✻ Stimulus | 0.00 | 1 | 182.00 | .962 |

| Parameter Estimates (Fixed coefficients) | | | | | | | | |
| --- | --- | --- | --- | --- | --- | --- | --- | --- |
|  | | | | 95% Confidence Intervals | |  | | |
| Names | Effect | Estimate | SE | Lower | Upper | df | t | p |
| (Intercept) | (Intercept) | 0.35 | 0.01 | 0.33 | 0.36 | 182.00 | 39.00 | <.001 |
| AOI1 | Mouth - Eyes | 0.27 | 0.03 | 0.21 | 0.32 | 182.00 | 9.39 | <.001 |
| Sync Condition1 | Async - Sync | 0.03 | 0.03 | -0.02 | 0.09 | 182.00 | 1.13 | .259 |
| Stimulus1 | Target - Distractor | 0.04 | 0.03 | -0.01 | 0.10 | 182.00 | 1.49 | .138 |
| AOI1 ✻ Sync Condition1 | (Mouth - Eyes) ✻ (Async - Sync) | 0.19 | 0.06 | 0.07 | 0.30 | 182.00 | 3.28 | .001 |
| AOI1 ✻ Stimulus1 | (Mouth - Eyes) ✻ (Target - Distractor) | 0.05 | 0.06 | -0.06 | 0.16 | 182.00 | 0.88 | .379 |
| Sync Condition1 ✻ Stimulus1 | (Async - Sync) ✻ (Target - Distractor) | -0.06 | 0.06 | -0.17 | 0.05 | 182.00 | -1.05 | .296 |
| AOI1 ✻ Sync Condition1 ✻ Stimulus1 | (Mouth - Eyes) ✻ (Async - Sync) ✻ (Target - Distractor) | -0.01 | 0.11 | -0.23 | 0.22 | 182.00 | -0.05 | .962 |

| Random Components | | | | | |
| --- | --- | --- | --- | --- | --- |
| Groups | Name | Variance | SD | ICC | rho |
| Subject | (Intercept) | 0.00 | 0.01 | 0.01 | -0.09 |
| Residual |  | 0.04 | 0.20 |  |  |
| Note. Number of Obs: 216 , Number of groups: Subject 27 | | | | | |

**Experiment 2**

**Cognitive Effort**

*LMM Analysis of Pupil Diameter*

**Mixed Model**

| Model Info | | |
| --- | --- | --- |
| Info |  |  |
| Model Type | Mixed Model | Linear Mixed model for continuous y |
| Model | lme | PupilSize ~ 1 + Trial + `Sync Condition` + Trial:`Sync Condition` + ( 1 \| Subject ) |
| Distribution | Gaussian | Normal distribution of residuals |
| Direction | y | Dependend variable scores |
| Residuals | Compound Symmetry | within cluster Subject |
| Sample size | 1120 |  |
| Converged | yes |  |
| Y transform | none |  |
| C.I. method | Wald |  |

**Model Results**

| Model Fit | | | | |
| --- | --- | --- | --- | --- |
| Type | R² | df | LRT X² | p |
| Conditional | 0.94 | 33 | 2699.90 | <.001 |
| Marginal | 0.02 | 31 | 138.17 | <.001 |

| Fixed Effects Omnibus Tests | | | | |
| --- | --- | --- | --- | --- |
|  | F | df | df (res) | p |
| Trial | 18.50 | 15 | 1054.00 | <.001 |
| Sync Condition | 28.11 | 1 | 1054.00 | <.001 |
| Trial ✻ Sync Condition | 0.93 | 15 | 1054.00 | .534 |

| Parameter Estimates (Fixed coefficients) | | | | | | | | |
| --- | --- | --- | --- | --- | --- | --- | --- | --- |
|  | | | | 95% Confidence Intervals | |  | | |
| Names | Effect | Estimate | SE | Lower | Upper | df | t | p |
| (Intercept) | (Intercept) | 4.68 | 0.13 | 4.42 | 4.93 | 1054.00 | 36.25 | <.001 |
| Trial1 | 2 - 1 | -0.10 | 0.03 | -0.16 | -0.03 | 1054.00 | -2.82 | .005 |
| Trial2 | 3 - 1 | -0.15 | 0.03 | -0.22 | -0.09 | 1054.00 | -4.48 | <.001 |
| Trial3 | 4 - 1 | -0.13 | 0.03 | -0.19 | -0.06 | 1054.00 | -3.72 | <.001 |
| Trial4 | 5 - 1 | -0.20 | 0.03 | -0.26 | -0.13 | 1054.00 | -5.74 | <.001 |
| Trial5 | 6 - 1 | -0.21 | 0.03 | -0.27 | -0.14 | 1054.00 | -6.03 | <.001 |
| Trial6 | 7 - 1 | -0.29 | 0.03 | -0.36 | -0.23 | 1054.00 | -8.60 | <.001 |
| Trial7 | 8 - 1 | -0.24 | 0.03 | -0.31 | -0.17 | 1054.00 | -7.01 | <.001 |
| Trial8 | 9 - 1 | -0.24 | 0.03 | -0.31 | -0.17 | 1054.00 | -7.05 | <.001 |
| Trial9 | 10 - 1 | -0.29 | 0.03 | -0.36 | -0.23 | 1054.00 | -8.57 | <.001 |
| Trial10 | 11 - 1 | -0.30 | 0.03 | -0.37 | -0.23 | 1054.00 | -8.81 | <.001 |
| Trial11 | 12 - 1 | -0.29 | 0.03 | -0.36 | -0.22 | 1054.00 | -8.46 | <.001 |
| Trial12 | 13 - 1 | -0.34 | 0.03 | -0.41 | -0.27 | 1054.00 | -9.94 | <.001 |
| Trial13 | 14 - 1 | -0.35 | 0.03 | -0.42 | -0.29 | 1054.00 | -10.29 | <.001 |
| Trial14 | 15 - 1 | -0.35 | 0.03 | -0.42 | -0.28 | 1054.00 | -10.28 | <.001 |
| Trial15 | 16 - 1 | -0.36 | 0.03 | -0.43 | -0.29 | 1054.00 | -10.47 | <.001 |
| Sync Condition1 | Async - Sync | 0.06 | 0.01 | 0.04 | 0.09 | 1054.00 | 5.30 | <.001 |
| Trial1 ✻ Sync Condition1 | (2 - 1) ✻ (Async - Sync) | 0.00 | 0.07 | -0.13 | 0.14 | 1054.00 | 0.06 | .948 |
| Trial2 ✻ Sync Condition1 | (3 - 1) ✻ (Async - Sync) | -0.02 | 0.07 | -0.15 | 0.11 | 1054.00 | -0.29 | .769 |
| Trial3 ✻ Sync Condition1 | (4 - 1) ✻ (Async - Sync) | -0.02 | 0.07 | -0.16 | 0.11 | 1054.00 | -0.32 | .747 |
| Trial4 ✻ Sync Condition1 | (5 - 1) ✻ (Async - Sync) | 0.01 | 0.07 | -0.12 | 0.15 | 1054.00 | 0.20 | .838 |
| Trial5 ✻ Sync Condition1 | (6 - 1) ✻ (Async - Sync) | -0.04 | 0.07 | -0.17 | 0.10 | 1054.00 | -0.58 | .565 |
| Trial6 ✻ Sync Condition1 | (7 - 1) ✻ (Async - Sync) | 0.05 | 0.07 | -0.08 | 0.19 | 1054.00 | 0.75 | .456 |
| Trial7 ✻ Sync Condition1 | (8 - 1) ✻ (Async - Sync) | -0.06 | 0.07 | -0.20 | 0.07 | 1054.00 | -0.94 | .345 |
| Trial8 ✻ Sync Condition1 | (9 - 1) ✻ (Async - Sync) | -0.07 | 0.07 | -0.21 | 0.06 | 1054.00 | -1.06 | .289 |
| Trial9 ✻ Sync Condition1 | (10 - 1) ✻ (Async - Sync) | 0.03 | 0.07 | -0.11 | 0.16 | 1054.00 | 0.39 | .697 |
| Trial10 ✻ Sync Condition1 | (11 - 1) ✻ (Async - Sync) | 0.02 | 0.07 | -0.11 | 0.16 | 1054.00 | 0.32 | .749 |
| Trial11 ✻ Sync Condition1 | (12 - 1) ✻ (Async - Sync) | -0.05 | 0.07 | -0.19 | 0.08 | 1054.00 | -0.75 | .456 |
| Trial12 ✻ Sync Condition1 | (13 - 1) ✻ (Async - Sync) | -0.14 | 0.07 | -0.27 | -0.00 | 1054.00 | -1.99 | .047 |
| Trial13 ✻ Sync Condition1 | (14 - 1) ✻ (Async - Sync) | 0.01 | 0.07 | -0.12 | 0.14 | 1054.00 | 0.14 | .886 |
| Trial14 ✻ Sync Condition1 | (15 - 1) ✻ (Async - Sync) | -0.01 | 0.07 | -0.15 | 0.12 | 1054.00 | -0.18 | .859 |
| Trial15 ✻ Sync Condition1 | (16 - 1) ✻ (Async - Sync) | 0.01 | 0.07 | -0.12 | 0.15 | 1054.00 | 0.17 | .869 |

| Random Components | | | | | |
| --- | --- | --- | --- | --- | --- |
| Groups | Name | Variance | SD | ICC | rho |
| Subject | (Intercept) | 0.58 | 0.76 | 0.93 | 0.00 |
| Residual |  | 0.04 | 0.20 |  |  |
| Note. Number of Obs: 1120 , Number of groups: Subject 35 | | | | | |

**Cross-Experiment Comparison: Experiments 1 vs. 2**

**Cognitive Effort***LMM Analysis of Pupil Diameter*

# **Mixed Model**

| Model Info | | |
| --- | --- | --- |
| Info |  |  |
| Model Type | Mixed Model | Linear Mixed model for continuous y |
| Model | lme | `Pupil Diameter` ~ 1 + Experiment + `Sync Condition` + Trial + Experiment:`Sync Condition` + Experiment:Trial + `Sync Condition`:Trial + Experiment:`Sync Condition`:Trial + ( 1 \| Subject ) |
| Distribution | Gaussian | Normal distribution of residuals |
| Direction | y | Dependend variable scores |
| Residuals | Compound Symmetry | within cluster Subject |
| Sample size | 2144 |  |
| Converged | yes |  |
| Y transform | none |  |
| C.I. method | Wald |  |

## **Model Results**

| Model Fit | | | | |
| --- | --- | --- | --- | --- |
| Type | R² | df | LRT X² | p |
| Conditional | 0.94 | 65 | 5283.00 | <.001 |
| Marginal | 0.07 | 63 | 102.05 | .001 |

| Fixed Effects Omnibus Tests | | | | |
| --- | --- | --- | --- | --- |
|  | F | df | df (res) | p |
| Experiment | 4.84 | 1 | 65.00 | .031 |
| Sync Condition | 20.04 | 1 | 2015.00 | <.001 |
| Trial | 19.54 | 15 | 2015.00 | <.001 |
| Experiment ✻ Sync Condition | 5.76 | 1 | 2015.00 | .016 |
| Experiment ✻ Trial | 2.05 | 15 | 2015.00 | .010 |
| Sync Condition ✻ Trial | 1.80 | 15 | 2015.00 | .030 |
| Experiment ✻ Sync Condition ✻ Trial | 1.91 | 15 | 2015.00 | .018 |

| Parameter Estimates (Fixed coefficients) | | | | | | | | |
| --- | --- | --- | --- | --- | --- | --- | --- | --- |
|  | | | | 95% Confidence Intervals | |  | | |
| Names | Effect | Estimate | SE | Lower | Upper | df | t | p |
| (Intercept) | (Intercept) | 4.46 | 0.10 | 4.26 | 4.65 | 2015.00 | 44.97 | <.001 |
| Experiment1 | 2 - 1 | 0.44 | 0.20 | 0.04 | 0.83 | 65.00 | 2.20 | .031 |
| Sync Condition1 | Async - Sync | 0.04 | 0.01 | 0.02 | 0.06 | 2015.00 | 4.48 | <.001 |
| Trial1 | 2 - 1 | -0.07 | 0.03 | -0.12 | -0.02 | 2015.00 | -2.58 | .010 |
| Trial2 | 3 - 1 | -0.11 | 0.03 | -0.17 | -0.06 | 2015.00 | -4.31 | <.001 |
| Trial3 | 4 - 1 | -0.11 | 0.03 | -0.17 | -0.06 | 2015.00 | -4.33 | <.001 |
| Trial4 | 5 - 1 | -0.16 | 0.03 | -0.21 | -0.11 | 2015.00 | -6.06 | <.001 |
| Trial5 | 6 - 1 | -0.19 | 0.03 | -0.24 | -0.14 | 2015.00 | -7.27 | <.001 |
| Trial6 | 7 - 1 | -0.23 | 0.03 | -0.28 | -0.18 | 2015.00 | -8.70 | <.001 |
| Trial7 | 8 - 1 | -0.21 | 0.03 | -0.26 | -0.16 | 2015.00 | -8.05 | <.001 |
| Trial8 | 9 - 1 | -0.20 | 0.03 | -0.25 | -0.15 | 2015.00 | -7.54 | <.001 |
| Trial9 | 10 - 1 | -0.25 | 0.03 | -0.31 | -0.20 | 2015.00 | -9.64 | <.001 |
| Trial10 | 11 - 1 | -0.25 | 0.03 | -0.30 | -0.20 | 2015.00 | -9.41 | <.001 |
| Trial11 | 12 - 1 | -0.25 | 0.03 | -0.30 | -0.20 | 2015.00 | -9.43 | <.001 |
| Trial12 | 13 - 1 | -0.27 | 0.03 | -0.32 | -0.22 | 2015.00 | -10.31 | <.001 |
| Trial13 | 14 - 1 | -0.28 | 0.03 | -0.33 | -0.23 | 2015.00 | -10.65 | <.001 |
| Trial14 | 15 - 1 | -0.26 | 0.03 | -0.31 | -0.21 | 2015.00 | -9.85 | <.001 |
| Trial15 | 16 - 1 | -0.27 | 0.03 | -0.32 | -0.22 | 2015.00 | -10.12 | <.001 |
| Experiment1 ✻ Sync Condition1 | (2 - 1) ✻ (Async - Sync) | 0.04 | 0.02 | 0.01 | 0.08 | 2015.00 | 2.40 | .016 |
| Experiment1 ✻ Trial1 | (2 - 1) ✻ (2 - 1) | -0.06 | 0.05 | -0.16 | 0.05 | 2015.00 | -1.08 | .281 |
| Experiment1 ✻ Trial2 | (2 - 1) ✻ (3 - 1) | -0.08 | 0.05 | -0.18 | 0.02 | 2015.00 | -1.49 | .135 |
| Experiment1 ✻ Trial3 | (2 - 1) ✻ (4 - 1) | -0.03 | 0.05 | -0.13 | 0.08 | 2015.00 | -0.50 | .619 |
| Experiment1 ✻ Trial4 | (2 - 1) ✻ (5 - 1) | -0.07 | 0.05 | -0.18 | 0.03 | 2015.00 | -1.39 | .166 |
| Experiment1 ✻ Trial5 | (2 - 1) ✻ (6 - 1) | -0.03 | 0.05 | -0.13 | 0.07 | 2015.00 | -0.56 | .575 |
| Experiment1 ✻ Trial6 | (2 - 1) ✻ (7 - 1) | -0.13 | 0.05 | -0.23 | -0.03 | 2015.00 | -2.46 | .014 |
| Experiment1 ✻ Trial7 | (2 - 1) ✻ (8 - 1) | -0.06 | 0.05 | -0.16 | 0.05 | 2015.00 | -1.05 | .294 |
| Experiment1 ✻ Trial8 | (2 - 1) ✻ (9 - 1) | -0.08 | 0.05 | -0.19 | 0.02 | 2015.00 | -1.60 | .111 |
| Experiment1 ✻ Trial9 | (2 - 1) ✻ (10 - 1) | -0.08 | 0.05 | -0.18 | 0.03 | 2015.00 | -1.48 | .140 |
| Experiment1 ✻ Trial10 | (2 - 1) ✻ (11 - 1) | -0.11 | 0.05 | -0.21 | -0.00 | 2015.00 | -2.02 | .044 |
| Experiment1 ✻ Trial11 | (2 - 1) ✻ (12 - 1) | -0.08 | 0.05 | -0.19 | 0.02 | 2015.00 | -1.54 | .123 |
| Experiment1 ✻ Trial12 | (2 - 1) ✻ (13 - 1) | -0.14 | 0.05 | -0.24 | -0.03 | 2015.00 | -2.57 | .010 |
| Experiment1 ✻ Trial13 | (2 - 1) ✻ (14 - 1) | -0.14 | 0.05 | -0.25 | -0.04 | 2015.00 | -2.69 | .007 |
| Experiment1 ✻ Trial14 | (2 - 1) ✻ (15 - 1) | -0.18 | 0.05 | -0.29 | -0.08 | 2015.00 | -3.48 | <.001 |
| Experiment1 ✻ Trial15 | (2 - 1) ✻ (16 - 1) | -0.18 | 0.05 | -0.29 | -0.08 | 2015.00 | -3.47 | <.001 |
| Sync Condition1 ✻ Trial1 | (Async - Sync) ✻ (2 - 1) | -0.05 | 0.05 | -0.15 | 0.05 | 2015.00 | -0.96 | .335 |
| Sync Condition1 ✻ Trial2 | (Async - Sync) ✻ (3 - 1) | -0.07 | 0.05 | -0.17 | 0.04 | 2015.00 | -1.25 | .211 |
| Sync Condition1 ✻ Trial3 | (Async - Sync) ✻ (4 - 1) | -0.06 | 0.05 | -0.17 | 0.04 | 2015.00 | -1.20 | .232 |
| Sync Condition1 ✻ Trial4 | (Async - Sync) ✻ (5 - 1) | -0.07 | 0.05 | -0.18 | 0.03 | 2015.00 | -1.41 | .158 |
| Sync Condition1 ✻ Trial5 | (Async - Sync) ✻ (6 - 1) | -0.05 | 0.05 | -0.15 | 0.05 | 2015.00 | -0.96 | .339 |
| Sync Condition1 ✻ Trial6 | (Async - Sync) ✻ (7 - 1) | 0.01 | 0.05 | -0.09 | 0.11 | 2015.00 | 0.19 | .849 |
| Sync Condition1 ✻ Trial7 | (Async - Sync) ✻ (8 - 1) | -0.05 | 0.05 | -0.16 | 0.05 | 2015.00 | -1.02 | .306 |
| Sync Condition1 ✻ Trial8 | (Async - Sync) ✻ (9 - 1) | -0.09 | 0.05 | -0.20 | 0.01 | 2015.00 | -1.75 | .081 |
| Sync Condition1 ✻ Trial9 | (Async - Sync) ✻ (10 - 1) | -0.08 | 0.05 | -0.18 | 0.03 | 2015.00 | -1.48 | .140 |
| Sync Condition1 ✻ Trial10 | (Async - Sync) ✻ (11 - 1) | -0.05 | 0.05 | -0.15 | 0.06 | 2015.00 | -0.87 | .386 |
| Sync Condition1 ✻ Trial11 | (Async - Sync) ✻ (12 - 1) | -0.10 | 0.05 | -0.20 | 0.01 | 2015.00 | -1.85 | .064 |
| Sync Condition1 ✻ Trial12 | (Async - Sync) ✻ (13 - 1) | -0.16 | 0.05 | -0.26 | -0.05 | 2015.00 | -2.99 | .003 |
| Sync Condition1 ✻ Trial13 | (Async - Sync) ✻ (14 - 1) | -0.11 | 0.05 | -0.22 | -0.01 | 2015.00 | -2.17 | .030 |
| Sync Condition1 ✻ Trial14 | (Async - Sync) ✻ (15 - 1) | -0.16 | 0.05 | -0.27 | -0.06 | 2015.00 | -3.12 | .002 |
| Sync Condition1 ✻ Trial15 | (Async - Sync) ✻ (16 - 1) | -0.14 | 0.05 | -0.25 | -0.04 | 2015.00 | -2.74 | .006 |
| Experiment1 ✻ Sync Condition1 ✻ Trial1 | (2 - 1) ✻ (Async - Sync) ✻ (2 - 1) | 0.11 | 0.11 | -0.10 | 0.32 | 2015.00 | 1.05 | .294 |
| Experiment1 ✻ Sync Condition1 ✻ Trial2 | (2 - 1) ✻ (Async - Sync) ✻ (3 - 1) | 0.09 | 0.11 | -0.12 | 0.30 | 2015.00 | 0.87 | .384 |
| Experiment1 ✻ Sync Condition1 ✻ Trial3 | (2 - 1) ✻ (Async - Sync) ✻ (4 - 1) | 0.08 | 0.11 | -0.13 | 0.29 | 2015.00 | 0.78 | .438 |
| Experiment1 ✻ Sync Condition1 ✻ Trial4 | (2 - 1) ✻ (Async - Sync) ✻ (5 - 1) | 0.18 | 0.11 | -0.03 | 0.38 | 2015.00 | 1.68 | .093 |
| Experiment1 ✻ Sync Condition1 ✻ Trial5 | (2 - 1) ✻ (Async - Sync) ✻ (6 - 1) | 0.02 | 0.11 | -0.19 | 0.23 | 2015.00 | 0.21 | .834 |
| Experiment1 ✻ Sync Condition1 ✻ Trial6 | (2 - 1) ✻ (Async - Sync) ✻ (7 - 1) | 0.08 | 0.11 | -0.13 | 0.29 | 2015.00 | 0.78 | .437 |
| Experiment1 ✻ Sync Condition1 ✻ Trial7 | (2 - 1) ✻ (Async - Sync) ✻ (8 - 1) | -0.02 | 0.11 | -0.23 | 0.19 | 2015.00 | -0.20 | .841 |
| Experiment1 ✻ Sync Condition1 ✻ Trial8 | (2 - 1) ✻ (Async - Sync) ✻ (9 - 1) | 0.04 | 0.11 | -0.17 | 0.25 | 2015.00 | 0.37 | .710 |
| Experiment1 ✻ Sync Condition1 ✻ Trial9 | (2 - 1) ✻ (Async - Sync) ✻ (10 - 1) | 0.21 | 0.11 | 0.00 | 0.42 | 2015.00 | 1.98 | .048 |
| Experiment1 ✻ Sync Condition1 ✻ Trial10 | (2 - 1) ✻ (Async - Sync) ✻ (11 - 1) | 0.14 | 0.11 | -0.07 | 0.34 | 2015.00 | 1.28 | .200 |
| Experiment1 ✻ Sync Condition1 ✻ Trial11 | (2 - 1) ✻ (Async - Sync) ✻ (12 - 1) | 0.09 | 0.11 | -0.11 | 0.30 | 2015.00 | 0.88 | .377 |
| Experiment1 ✻ Sync Condition1 ✻ Trial12 | (2 - 1) ✻ (Async - Sync) ✻ (13 - 1) | 0.04 | 0.11 | -0.16 | 0.25 | 2015.00 | 0.41 | .684 |
| Experiment1 ✻ Sync Condition1 ✻ Trial13 | (2 - 1) ✻ (Async - Sync) ✻ (14 - 1) | 0.25 | 0.11 | 0.04 | 0.46 | 2015.00 | 2.35 | .019 |
| Experiment1 ✻ Sync Condition1 ✻ Trial14 | (2 - 1) ✻ (Async - Sync) ✻ (15 - 1) | 0.31 | 0.11 | 0.10 | 0.51 | 2015.00 | 2.89 | .004 |
| Experiment1 ✻ Sync Condition1 ✻ Trial15 | (2 - 1) ✻ (Async - Sync) ✻ (16 - 1) | 0.31 | 0.11 | 0.10 | 0.52 | 2015.00 | 2.95 | .003 |

| Random Components | | | | | |
| --- | --- | --- | --- | --- | --- |
| Groups | Name | Variance | SD | ICC | rho |
| Subject | (Intercept) | 0.66 | 0.81 | 0.93 | 0.00 |
| Residual |  | 0.05 | 0.22 |  |  |
| Note. Number of Obs: 2144 , Number of groups: Subject 67 | | | | | |

**Cross-Study Comparisons: Eye and Mouth AOIs in Experiment 1 vs. Experiment 1 in Lewkowicz et al. (1)**

*LMM Analysis of PTLT Scores*

# **Mixed Model**

| Model Info | | |
| --- | --- | --- |
| Info |  |  |
| Model Type | Mixed Model | Linear Mixed model for continuous y |
| Model | lme | PTLT ~ 1 + Experiment + AOI + Stimulus + Experiment:AOI + Experiment:Stimulus + AOI:Stimulus + Experiment:AOI:Stimulus + ( 1 \| Subject ) |
| Distribution | Gaussian | Normal distribution of residuals |
| Direction | y | Dependend variable scores |
| Residuals | Compound Symmetry | within cluster Subject |
| Sample size | 268 |  |
| Converged | yes |  |
| Y transform | none |  |
| C.I. method | Wald |  |

## **Model Results**

| Model Fit | | | | |
| --- | --- | --- | --- | --- |
| Type | R² | df | LRT X² | p |
| Conditional | 0.55 | 9 | 183.98 | <.001 |
| Marginal | 0.54 | 7 | 143.09 | <.001 |

| Fixed Effects Omnibus Tests | | | | |
| --- | --- | --- | --- | --- |
|  | F | df | df (res) | p |
| Experiment | 3.61 | 1 | 65.00 | .062 |
| AOI | 232.13 | 1 | 195.00 | <.001 |
| Stimulus | 18.93 | 1 | 195.00 | <.001 |
| Experiment ✻ AOI | 13.49 | 1 | 195.00 | <.001 |
| Experiment ✻ Stimulus | 0.55 | 1 | 195.00 | .460 |
| AOI ✻ Stimulus | 2.65 | 1 | 195.00 | .105 |
| Experiment ✻ AOI ✻ Stimulus | 14.53 | 1 | 195.00 | <.001 |

| Parameter Estimates (Fixed coefficients) | | | | | | | | |
| --- | --- | --- | --- | --- | --- | --- | --- | --- |
|  | | | | 95% Confidence Intervals | |  | | |
| Names | Effect | Estimate | SE | Lower | Upper | df | t | p |
| (Intercept) | (Intercept) | 0.31 | 0.01 | 0.29 | 0.32 | 195.00 | 39.77 | <.001 |
| Experiment1 | 2 - 1 | 0.03 | 0.02 | -0.00 | 0.06 | 65.00 | 1.90 | .062 |
| AOI1 | mouth - eyes | 0.36 | 0.02 | 0.31 | 0.40 | 195.00 | 15.24 | <.001 |
| Stimulus1 | Target - Distractor | 0.10 | 0.02 | 0.06 | 0.15 | 195.00 | 4.35 | <.001 |
| Experiment1 ✻ AOI1 | (2 - 1) ✻ (mouth - eyes) | 0.17 | 0.05 | 0.08 | 0.26 | 195.00 | 3.67 | <.001 |
| Experiment1 ✻ Stimulus1 | (2 - 1) ✻ (Target - Distractor) | 0.03 | 0.05 | -0.06 | 0.13 | 195.00 | 0.74 | .460 |
| AOI1 ✻ Stimulus1 | (mouth - eyes) ✻ (Target - Distractor) | 0.08 | 0.05 | -0.02 | 0.17 | 195.00 | 1.63 | .105 |
| Experiment1 ✻ AOI1 ✻ Stimulus1 | (2 - 1) ✻ (mouth - eyes) ✻ (Target - Distractor) | 0.36 | 0.09 | 0.17 | 0.54 | 195.00 | 3.81 | <.001 |

| Random Components | | | | | |
| --- | --- | --- | --- | --- | --- |
| Groups | Name | Variance | SD | ICC | rho |
| Subject | (Intercept) | 0.00 | 0.02 | 0.02 | -0.18 |
| Residual |  | 0.03 | 0.17 |  |  |
| Note. Number of Obs: 268 , Number of groups: Subject 67 | | | | | |

**Cross-Study Comparisons: Face AOIs in Experiment 2 vs. Experiment 2 in Lewkowicz et al. (1)**

*LMM Analysis of PTLT Scores*

# **Mixed Model**

| Model Info | | |
| --- | --- | --- |
| Info |  |  |
| Model Type | Mixed Model | Linear Mixed model for continuous y |
| Model | lme | PTLT ~ 1 + Stimulus + `Sync Condition` + Experiment + Stimulus:`Sync Condition` + Stimulus:Experiment + `Sync Condition`:Experiment + Stimulus:`Sync Condition`:Experiment + ( 1 \| Subject ) |
| Distribution | Gaussian | Normal distribution of residuals |
| Direction | y | Dependend variable scores |
| Residuals | Compound Symmetry | within cluster Subject |
| Sample size | 232 |  |
| Converged | yes |  |
| Y transform | none |  |
| C.I. method | Wald |  |

## **Model Results**

| Model Fit | | | | |
| --- | --- | --- | --- | --- |
| Type | R² | df | LRT X² | p |
| Conditional | 0.90 | 9 | 506.35 | <.001 |
| Marginal | 0.90 | 7 | 400.62 | <.001 |

| Fixed Effects Omnibus Tests | | | | |
| --- | --- | --- | --- | --- |
|  | F | df | df (res) | p |
| Stimulus | 1271.32 | 1 | 168.00 | <.001 |
| Sync Condition | 106.70 | 1 | 168.00 | <.001 |
| Experiment | 9.97 | 1 | 56.00 | .003 |
| Stimulus ✻ Sync Condition | 434.26 | 1 | 168.00 | <.001 |
| Stimulus ✻ Experiment | 18.23 | 1 | 168.00 | <.001 |
| Sync Condition ✻ Experiment | 3.12 | 1 | 168.00 | .079 |
| Stimulus ✻ Sync Condition ✻ Experiment | 13.20 | 1 | 168.00 | <.001 |

| Parameter Estimates (Fixed coefficients) | | | | | | | | |
| --- | --- | --- | --- | --- | --- | --- | --- | --- |
|  | | | | 95% Confidence Intervals | |  | | |
| Names | Effect | Estimate | SE | Lower | Upper | df | t | p |
| (Intercept) | (Intercept) | 0.36 | 0.00 | 0.35 | 0.37 | 168.00 | 83.59 | <.001 |
| Stimulus1 | Target - Distractor | 0.44 | 0.01 | 0.42 | 0.47 | 168.00 | 35.66 | <.001 |
| Sync Condition1 | Async - Sync | -0.13 | 0.01 | -0.15 | -0.10 | 168.00 | -10.33 | <.001 |
| Experiment1 | 3 - 2 | 0.03 | 0.01 | 0.01 | 0.04 | 56.00 | 3.16 | .003 |
| Stimulus1 ✻ Sync Condition1 | (Target - Distractor) ✻ (Async - Sync) | -0.52 | 0.02 | -0.57 | -0.47 | 168.00 | -20.84 | <.001 |
| Stimulus1 ✻ Experiment1 | (Target - Distractor) ✻ (3 - 2) | 0.11 | 0.02 | 0.06 | 0.16 | 168.00 | 4.27 | <.001 |
| Sync Condition1 ✻ Experiment1 | (Async - Sync) ✻ (3 - 2) | 0.04 | 0.02 | -0.01 | 0.09 | 168.00 | 1.77 | .079 |
| Stimulus1 ✻ Sync Condition1 ✻ Experiment1 | (Target - Distractor) ✻ (Async - Sync) ✻ (3 - 2) | 0.18 | 0.05 | 0.08 | 0.28 | 168.00 | 3.63 | <.001 |

| Random Components | | | | | |
| --- | --- | --- | --- | --- | --- |
| Groups | Name | Variance | SD | ICC | rho |
| Subject | (Intercept) | 0.00 | 0.01 | 0.02 | -0.17 |
| Residual |  | 0.01 | 0.09 |  |  |
| Note. Number of Obs: 232 , Number of groups: Subject 58 | | | | | |
